# The role of exosomes for sustained specific cardiorespiratory and metabolic improvements in males with type 2 diabetes after detraining

**DOI:** 10.1016/j.ebiom.2024.105471

**Published:** 2024-12-02

**Authors:** Lucia Mastrototaro, Maria Apostolopoulou, Sonja Hartwig, Klaus Strassburger, Polina Lipaeva, Nina Trinks, Yanislava Karusheva, Sofiya Gancheva, Sandra Trenkamp, Stefan Lehr, Hadi Al-Hasani, Julia Szendroedi, Michael Roden

**Affiliations:** a Institute for Clinical Diabetology, German Diabetes Center, Leibniz Center for Diabetes Research at Heinrich-Heine University Düsseldorf, Düsseldorf, Germany; b German Center for Diabetes Research, Partner Düsseldorf, München-Neuherberg, Germany; c Department of Endocrinology and Diabetology, Medical Faculty and University Hospital, Heinrich-Heine-University, Düsseldorf, Germany; d Institute for Clinical Biochemistry and Pathobiochemistry German Diabetes Center, Leibniz Center for Diabetes Research at Heinrich-Heine University Düsseldorf, Düsseldorf, Germany; e Institute for Biometrics and Epidemiology, German Diabetes Center, Leibniz Center for Diabetes Research at Heinrich-Heine University Düsseldorf, Düsseldorf, Germany; f Institute for Medical Biometry and Bioinformatics, Medical Faculty and University Hospital, Heinrich Heine University, Düsseldorf, Germany; g Department of Medicine I and Clinical Chemistry, University Hospital of Heidelberg, Heidelberg, Germany

**Keywords:** Exercise, Detraining, Insulin sensitivity, Small extracellular vesicles, Inter-organ crosstalk

## Abstract

**Background:**

High-intensity interval training (HIIT) has been shown to improve cardiorespiratory fitness ($\dot{V}$O_2_ max) but may ameliorate insulin sensitivity only in insulin-resistant humans. It is yet unclear whether these benefits persist after detraining and to which extent duration and effectiveness of metabolic improvements differ between individuals without and with prediabetes or type 2 diabetes (T2D). Understanding these differences is relevant for developing targeted exercise training modes for individuals with different stages of dysglycemia.

**Methods:**

Men with (20 T2D) and without T2D (12 insulin-sensitive, IS-NDM; 10 insulin-resistant, IR-NDM) underwent hyperinsulinemic-euglycemic clamps, spiroergometry, ectopic lipid quantification and muscle biopsies at baseline, after 12-week HIIT and after 4-week detraining.

**Findings:**

After detraining, the HIIT-stimulated $\dot{V}$O_2_ max declined in T2D and IR-NDM, but remained higher compared to baseline in all groups. The HIIT-induced changes in hepatic insulin sensitivity and ectopic lipid content were sustained after detraining in T2D and IR-NDM, whereas improvements of whole-body insulin sensitivity were abolished in T2D. T2D and IR-NDM showed persistent increases in the number of small extracellular vesicles, which carry among others antioxidant proteins. The ratio of reduced-to-oxidized glutathione further decreased after detraining in all groups, whereas changes in proteins involved in mitochondrial turnover were dependent on insulin sensitivity, with some evidence for upregulation of fusion and mitophagy in T2D and IR-NDM and upregulation of fission in IS-NDM. Levels of different lipolytic proteins were reduced in all participants after detraining.

**Interpretation:**

HIIT offers sustained improvement of energy metabolism and hepatic insulin sensitivity in insulin-resistant humans, but long-term adherence is required to maintain these benefits.

**Funding:**

Funding bodies that contributed to this study are listed in the Acknowledgements section.

**Research in context**

**Evidence before this study**

High-intensity interval training (HIIT) represents a highly efficient alternative to moderate training modalities and may exert superior benefits for enhancing cardiorespiratory fitness and insulin sensitivity in humans without or with prediabetes or type 2 diabetes (T2D). The beneficial effects are at least partly mediated by inter-organ communication via small extracellular vesicles (SEV), but the sustainability of the positive effects after stopping HIIT training in healthy and glucose-intolerant individuals are yet unclear.

**Added value of this study**

This study examined whether the HIIT-induced metabolic benefits are maintained or differ between individuals with and without T2D following an extended period of training cessation. Whole-body and hepatic insulin sensitivity, ectopic lipid contents, features of muscle mitochondrial function and lipid metabolism were assessed at baseline, after 12-week HIIT, and after 4-week detraining in individuals with different degrees of glucose tolerance. Further, the study investigated the number and protein cargo of circulating SEV and their possible contribution to sustained metabolic changes observed after detraining.

**Implications of all the available evidence**

While HIIT-induced increases in whole-body oxidative metabolism and insulin sensitivity diminish after 4 weeks of detraining in insulin-resistant individuals and in T2D respectively, the improvements in hepatic lipid content and hepatic insulin sensitivity persist. The observed metabolic changes are at least partly due to changes in SEV number and protein levels (i.e., PRDX2, GPX3, PTGS1, PSMB3, CARD9, MPO), supporting their possible role as mediators of exercise-induced inter-organ communication.

## Introduction

Moderate aerobic exercise training improves insulin sensitivity and mitochondrial functionality in people without or with type 2 diabetes (T2D).^1^ These improvements primarily occur through induction of mitochondrial biogenesis,^2^ but also fusion and fission,^1^^,^^3^ altering the mitochondria network organization.^4^ Furthermore, moderate exercise training induces mitophagy in skeletal muscle of healthy individuals.^5^ Exercise training also induces upregulation of the lipolytic proteins, adipose triglyceride lipase (ATGL), and hormone sensitive lipase (HSL), in skeletal muscle which results in augmented lipid mobilization, reduced intramuscular triglyceride content, and increased fatty acid oxidation.^6^^,^^7^ On the other hand, improved diacylglycerol acyltransferase (DGAT1) activity results in elimination of intramyocellular diacylglycerol (DAG) and improved insulin sensitivity.^8^ Indeed, DAG is an important mediator of insulin resistance interfering with insulin signaling by activation of novel protein kinase (nPKC) θ.^9^ Termination of conventional exercise training (detraining) for 4–8 weeks completely reverses the improvements in insulin sensitivity and mitochondrial functionality in individuals without^10^ or with the metabolic syndrome.^11^ Interruption of combined aerobic and strength training for 12 weeks further abolishes the beneficial effects on body composition, glycemic control and physical fitness in women with T2D.^12^ Also intensively-trained athletes cannot maintain higher insulin sensitivity, $\dot{V}$O_2_ max and ATP production after short-term detraining,^13^ while sedentary lean^14^ or obese individuals^15^ do not immediately reverse higher insulin sensitivity at 2-weeks after high-volume/vigorous-intensity training. Finally, the interruption of physical activity reduces the expression levels of DGAT1 and increases the levels of ATGL in skeletal muscle of mice with normal or high-fat diet, resulting in DAG accumulation and impaired insulin sensitivity.^16^

High-intensity interval training (HIIT) is a time-efficient exercise modality that improves cardiorespiratory fitness and insulin sensitivity in people without, with or at risk of T2D.^17^^,^^18^ HIIT specifically increases citrate synthase activity, mitochondrial capacity, biogenesis and/or fusion as well as lipolysis in individuals without^19^, ^20^, ^21^, ^22^ or with T2D.^23^ Our recent study showed that HIIT indeed uniformly increases muscle mitochondrial capacity in humans independent of their insulin sensitivity, but improves whole-body insulin sensitivity mainly in insulin-resistant individuals.^24^ This was associated with lower activity of nPKC isoforms and reduced nuclear factor κ light chain enhancer of activated B cells (NF-κB) protein levels in muscle of insulin-resistant humans with or without T2D, along with specific changes in the number and protein cargo of circulating small extracellular vesicles (SEV).^24^

These observations were made at 72 h following the last bout of 12-week HIIT, raising the question about longer-term sustainability of the beneficial HIIT effects. Some studies already showed that certain effects are maintained after detraining for 2 and 4 weeks upon completion of 6- and 8-week HIIT in healthy^19^^,^^25^ and glucose-intolerant individuals.^18^ The aim of the present study was to compare the sustainability of metabolic effects induced by a longer-term HIIT between people with different degrees of whοle-body insulin sensitivity and people with overt T2D upon detraining. To this end, we included volunteers with and without T2D, who had performed a 12-week HIIT program^24^ followed by a 4-week detraining period and underwent detailed phenotyping (spiroergometry, hyperinsulinemic-euglycemic clamps with stable isotope dilution, ectopic lipid quantification, skeletal muscle biopsies) before and after training as well as after detraining. We hypothesized that the beneficial cardiometabolic effects on whole-body oxidative metabolism and insulin sensitivity, as well as hepatic insulin sensitivity and lipid contents, and the HIIT-induced changes in number and protein cargo of circulating SEV are maintained after detraining across all participants.

## Methods

### Study participants and design

This is a monocentric study, performed at the Clinical Research Center (CRC) of the German Diabetes Center (DDZ). The design of the intervention study (HIIT) has been described before.^24^ Briefly, all volunteers meeting the inclusion criteria, underwent comprehensive screening before entering the 12-week supervised cycle ergometer training protocol. Exclusion criteria included acute infections, history of malignancy or family history of T2D, thyroid dysfunction, smoking, reported relevant alcohol intake (>30 g/day) or drug abuse, night-shift working, medication with thiazolidinediones or beta-blockers, systematic endurance training (>60 min once weekly). All participants without T2D underwent a 75-g oral glucose tolerance test (OGTT) to include only those volunteers with euglycemic fasting (<100 mg/dl) and 2-h (<140 mg/dl) plasma glucose concentrations. Because of significant cardiometabolic and endocrine sex differences, the design allowed to include only one sex to ensure a homogenous study population. A priori sample size was calculated based on data from a previous study in our center,^26^ in order to determine the probability of detecting a 20% relative change in M-value with a power of 80% and a cut-off of p < 0.05 (significance level is adjusted to 0.025 according to the Bonferroni correction). Thus, this study included 20 males with T2D as well as 22 age-similar glucose-tolerant males (NDM) of comparable age, divided into 2 subgroups: 12 insulin-sensitive (IS-NDM) and 11 insulin-resistant (IR-NDM).^24^ IS-NDM were separated from IR-NDM using a cutoff M-value of >5.5 mg × kg^−1^ × min^−1^, following a previous publication.^27^ Age restriction was achieved by recruiting participants within a narrow age range of 50–60 years, ensuring comparability across the groups. The HIIT training consisted of 4-min high-intensity (exercising at 90% of the maximal heart rate) interspersed by three 3-min training intervals (exercising at 70% of the maximal heart rate). The total training duration was 35 min per session.

All but one IR-NDM participated in the subsequent 4-week detraining study (Supplementary Fig. S1). Humans with T2D were drug-naïve (n = 3), on metformin (n = 9), sulfonylureas (n = 2), metformin/DPP-4 inhibitor combination (n = 5) or metformin/basal insulin combination (n = 1). Participants received instructions to maintain stable body weight (5% of the initial body weight), and to decrease their physical activities down to the levels before the HIIT program during detraining. Body weight was recorded during weekly visits at DDZ and a change of 3% of the initial body weight during the intervention as well as at detraining resulted in dietary counseling by a clinical nutritionist. Physical activity levels were monitored using the international physical activity questionnaire (IPAQ).^28^ The study design is summarized in Supplementary Fig. S2. All participants gave written informed consent prior to inclusion in the study, which was approved by the institutional review board of Heinrich-Heine University Düsseldorf (NCT02039934) and performed according to the World's Medical Association Declaration of Helsinki.

### Hyperinsulinemic-euglycemic clamp test

Two-step hyperinsulinemic-euglycemic clamp tests for assessment of peripheral and hepatic insulin sensitivity were performed after a 10-h overnight fast one week before first training session (baseline), at 72 h after the last training session and following detraining. Participants with T2D refrained from their oral glucose lowering medication for three days before the clamp and/or injected their last basal insulin dose at nighttime, 12 h prior to the clamp.^21^ On the day of the clamp, participants arrived at the CRC at 7:00 a.m., and received two venous catheters in the antecubital veins of both arms for blood sampling and infusions of glucose and insulin. At −120 min a primed-continuous infusion of 98%-enriched D-[6,6-^2^H_2_] glucose was initiated and continued until the end of the clamp (+240 min) to measure endogenous glucose production (EGP). After zero time (0 min), insulin (Actrapid; Novo Nordisk, Copenhagen, Denmark) was given as a low-dose primed-continuous infusion (20 mU × m^2^ body surface area^−1^ × min^−1^) followed by a higher dose primed-continuous infusion (40 mU × m^2^ body surface area^−1^ × min^−1^) from 120 to 240 min.^24^^,^^29^ A 20% dextrose infusion labeled with D-[6,6-^2^H_2_] glucose (2% enrichment) was adjusted to maintain constant normoglycemia (target blood glucose: 90 mg/dl) and plasma enrichment of labeled glucose (2%). The M-value was calculated during the last 20 min of the clamp test (steady state) to assess whole-body insulin sensitivity, while hepatic insulin sensitivity was calculated as suppression of EGP by insulin during clamp (iEGP).

### Spiroergometry

All participants performed at baseline, after end of training program as well as following the detraining period an incremental exhaustive exercise test on an electronically-braked ergometer (Ergoline ergometrics 900, Bitz Germany). Respiratory gas exchange was monitored breath-by-breath for 12–16 min by open-air spirometry (Masterscreen CPX; Jäger/Viasys, Hoechberg, Germany) to obtain maximal rate of oxygen uptake ($\dot{V}$O_2_ max).^24^^,^^30^ $\dot{V}$O_2_ max was expressed in ml O_2_ × kg body weight^−1^ × min^−1^. Spiroergometry was performed three days prior to hyperinsulinemic-euglycemic clamp tests after a 10-h overnight fast.

### Oral glucose tolerance tests

During screening all participants without known T2D underwent a standard 2-h 75 g OGTT with fasting and 2-h blood sampling for measurement of plasma glucose. Additionally, on the day before spiroergometry, all participants with and without T2D underwent a 3-h OGTT at three time points: baseline, after 12-week HIIT and after 4 weeks of detraining for assessment of postprandial glucose metabolism, plasma blood glucose at 2 h and 3 h of OGTT (2-h PBG, 3-h PBG).

### Magnetic resonance imaging (MRI) and proton magnetic resonance spectroscopy (^1^H-MRS)

Whole-body, subcutaneous and visceral adipose tissue volume were measured by MRI, while liver lipid content was quantified by volume-selective ^1^H-MRS.^31^^,^^32^

### Skeletal muscle biopsy

Before the start of the clamp, skeletal muscle tissue (100–500 mg) was obtained from the *musculus vastus lateralis* and was used for high-resolution respirometry (HRR) or stored in liquid nitrogen and frozen at −80 °C until further analysis.^24^

### High-resolution respirometry (HRR)

*Ex vivo* analysis of mitochondrial respiration was performed in duplicate in permeabilized fibers from skeletal muscle biopsies (2–3 mg) using a HRR (Oxygraph-2k [O2k], Oroboros Instruments, Innsbruck, Austria), as described.^24^ Leak respiration with electron input through complex I (CI, CI_L_) was determined after addition of pyruvate (10 mM) and glutamate (10 mM), followed by injection of ADP (2.5 mM) to determine oxidative phosphorylation (OXPHOS) capacity through CI (CI_P_); succinate was then added (10 mM) to determine maximal OXPHOS capacity with convergent electron input through CI and CII (CI + II_P_) combined. Cytochrome *c* was added to test the integrity of the outer mitochondrial membrane, and chambers presenting an increase greater than 15% were excluded. Oxygen flux rates were corrected for tissue wet weight and are expressed as pmol O_2_ × mg wet weight^−1^ × s^−1^.

### Western blotting

Expression levels of proteins of interest were assessed by Western blotting as previously described.^24^ Briefly, proteins were extracted from approximately 30 mg of frozen skeletal muscle and homogenized in 300 μl of lysis buffer [25 mM tris-hydrochloride (tris–HCl), 1 mM Ethylenediaminetetraacetic acid (EDTA), 150 mM NaCl, and 0.20% NP-40] with protease (cOmplete Tablets, EASYpack, Roche Diagnostics, Basel, Switzerland) and phosphatase (PhosSTOP, EASYpack, Roche Diagnostics) inhibitors. Samples were shaken thrice for 1 min at 20 Hz in a Tissue Lyzer and centrifuged (13,000 rpm, 15 min, 4 °C) to pellet insolubilized material.

Aliquots of extracted proteins (30 μg) were diluted with reducing Laemmli sample buffer containing 2-mercaptoethanol (1610747, Bio-Rad, CA, USA), boiled 5 min at 95 °C and loaded onto a SDS–polyacrylamide gradient gel (4–20% Mini-PROTEAN TGX Precast Protein Gels, Bio-Rad, CA, USA). Following electrophoresis, proteins were transferred to a polyvinylidene difluoride membrane using the Trans-Blot Turbo Transfer System (Bio-Rad, CA, USA). After blocking the membranes for 2 h at room temperature (RT) using the blocking solution (5% milk in tris-buffered saline with Tween, TBST), we incubated the membranes overnight with primary antibodies at 4 °C (Supplementary Table S1). In this study, we used only commercially available antibodies selected according to the manufacturers’ documentation for Western blotting, for which no additional validation was performed. Next day, membranes were washed with TBST buffer and incubated with horseradish peroxidase-conjugated secondary antibodies for 1 h at RT (Supplementary Table S1). Last, we coated the membranes with Immobilon Western Chemiluminescent HRP substrate (Millipore, Danvers, MA, USA), and we detected the proteins using a Bio-Rad ChemiDoc MP Imaging System in combination with the software ImageLab 6.0.1 (Bio-Rad, CA, USA) for densitometric analysis. Data are expressed in arbitrary units and normalized either to GAPDH as reference loading control or to total protein content. For analysis and comparison of samples on different gels, an inter-run calibrator (IRC) was loaded as reference sample on each gel to correct for run-to-run variation (Supplemental Western Blots File).^33^

### Laboratory measurements

All blood analyses were performed after 10-h overnight fasting in the morning prior to the spiroergometry test with established methods as described previously.^24^ In aliquots of skeletal muscle (15 mg), total glutathione and oxidized glutathione (GSSG) contents were measured colorimetrically (Gluthathione Colorimetric Detection Kit; Thermo Fisher Scientific, Dreieich, Germany) based on the method of Griffith^34^ and normalized to protein concentration, quantified in the supernatant using the BCA Assay Kit (Thermo Fisher Scientific, Dreieich, Germany).^24^

### Small extracellular vesicles isolation and quantification

In aliquots of 0.5 ml serum, SEV were isolated by size exclusion chromatography (SEC), using the columns (qEV2/70 nm) from Izon Science (Addington, New Zealand) coupled to successive ultracentrifugation.^24^ Briefly, we cleared the serum samples at 1500*g* for 10 min followed by centrifugation at 10,000*g* for 10 min to remove particulate matter. Then, we loaded the serum supernatant into an IZON column previously equilibrated with phosphate-buffered saline (PBS). Once the serum entered the column, 50 ml of PBS was loaded on top of the column. The first 13 ml of flow-through was discarded, and the elution fractions of 14–21 ml were collected. Although serum proteins, such as albumin, apolipoproteins, and immunoglobulins, copurify with all isolation methods, SEC proved to efficiently isolate SEV fractions, as shown in transmission electron microscopy and western blot by the presence of typical EV markers and the absence of albumin and proteins associated with other intracellular compartment than plasma membrane.^24^ A total of 300 μl of PBS-eluted SEV were used for Nanoparticles Tracking Analysis, as described,^24^ while the rest was centrifuged at 100,000*g* for 70 min for mass spectrometry (MS) analysis.

### Mass spectrometry

Extracellular vesicles were prepared and measured by MS using an Orbitrap Exploris 480 system (ThermoFisher Scientific, Dreieich, Germany) according to the previously described procedure.^24^ Briefly, SEV pellets derived from human serum samples were lysed in denaturing SDS (sodium dodecyl sulfate) buffer (62.5 mM tris–HCl pH 6.8, 10% glycerol, 2 mM EDTA, 2% SDS, and 100 mM dithiothreitol (DTT)) and loaded onto SDS-PAGE (10% polyacrylamide, 0.5 cm separation distance). Coomassie blue stained protein bands were excised and subjected to tryptic in-gel protein digestion. For MS analysis, lyophilized peptides were reconstituted in 1% trifluoroacetic acid (TFA) (v/v) and separated by liquid chromatography (Ultimate 3000, Thermo Fisher Scientific, Dreieich, Germany) using an EASYspray ion source equipped to an Orbitrap Exploris 480 mass spectrometer (Thermo Fisher Scientific, Dreieich, Germany). Peptides were trapped and desalted on an Acclaim PepMap C18-LC-column (ID: 75 μm, 2 cm length; Thermo Fisher Scientific, Dreieich, Germany) and subsequently separated via EASY-Spray C18 column (ES803A, 50 cm × 75 μm inner diameter; Thermo Fisher Scientific, Dreieich, Germany) using a 100 min linear gradient from buffer A (0.1% formic acid) to 4–34% buffer B (80% acetonitrile, 0.1% formic acid) at a flow rate of 300 nl/min followed by a 20 min linear gradient increasing buffer B to 50% and a 1 min linear gradient increasing buffer B to 90%. Column temperature was set to 40 °C. MS data for label free quantification were acquired in a DIA (data independent acquisition) mode. Full scan MS spectra were obtained at 120,000 resolution, *m*/*z* range of 400–1200, and an AGC (automatic gain control) target value of 125% and maximum injection time of 50 ms. Fragmentation was performed with HCD (higher-energy collisional dissociation) energy of 32% in 34 windows cover the range from 400 to 1200 with a segment width of 24.5 (*m*/*z*), Orbitrap resolution of 30,000, AGC Target of 2000%, scan range from 200 to 2000 (*m*/*z*) and maximal injection time was 60 ms.

### Analysis of mass spectrometry data

For analysis, Pulsar search (Spectronaut Pulsar, Version 15, Biognosys, Schlieren, Switzerland) of DIA data was done against a human FASTA file (UniProt reference proteome, *Homo sapiens* TaxID 9096, proteome UP000005640, version 06-2021). Results were filtered by a false discovery rate (FDR) of 1% on precursor and protein group level (q-value < 0.01). For quantitative analysis, the DIA data were analyzed in Spectronaut (Version 15, Biognosys, Schlieren, Switzerland) using an in-house sample specific library^24^ supplemented by DIA runs with default settings. Candidates list was created using an absolute average log2 ratio ≥ 0.58 and q-value ≤ 0.05.

### Bioinformatic analysis

The protein information was extracted from Swiss-Prot database (RRID: SCR_021164). The prediction of a secretory signal peptide (SP) was performed with the server SignalP 4.1 (RRID: SCR_015644) using as cutoff 0.450, and the proteins without a SP were further analyzed with SecretomeP 2.0 to identify non classically secreted proteins (score > 0.6).^24^ The GO enrichment analysis for cellular composition (GO-CC), molecular function (GO-MF), and biological processes (GO-BP) of the SEV proteins was performed using the R package topGO version 2.42.0 (RRID: SCR_014798).^35^ The “algorithm” parameter was set to “classic”. Accessions for the corresponding GO terms were obtained from the EBI Gene Ontology Annotation Database (release data: 2023-04-01). The reported p-values (Fisher's exact test) were adjusted using the false discovery rate procedure (Benjamini–Hochberg correction, B–H). Enrichment analysis of the entire proteome dataset obtained in this study utilized a background list comprising all human genes, whereas for the enrichment analysis involving differentially abundant proteins between the groups, the list of proteins detected in this study was employed as the background. Only GO terms with B–H FDR ≤0.15 were considered significant and used to elucidate the responses observed within the groups (Supplementary Tables S2 and S3). R package ComplexHeatmap version 2.16.0^36^ was used to build heatmaps of differentially abundant proteins (absolute average log2 ratio ≥ 0.58 and q-value ≤ 0.01). Finally, Pearson as well as Spearman correlation coefficient (R and R_s_, respectively) were calculated to measure the relationship between individual SEV proteins and metabolic changes after detraining across all groups combined (T2D, IR-NDM, IS-NDM).

### Statistical analysis

Data are given as mean and standard error of mean (±SEM) or median (1st and 3rd quartile), as appropriate. All variables except age and body mass index (BMI) were log-transformed before analysis to approximate normality. This decision was primarily based on prior knowledge from other studies with larger sample size.^37^, ^38^, ^39^ Additionally, the fit of normal and log-normal distributions was visually assessed using cumulative distribution functions, and the log-normal distribution consistently showed a better fit than the normal distribution. In order to investigate changes between the different time points (baseline, 12-week HIIT and detraining) we performed linear mixed models analysis. This analysis was performed separately for each group (T2D, NDM, IR-NDM, IS-NDM). The dependency structure between time points was modeled via a compound symmetry or, equivalently, a random intercept model. Differences between groups at baseline were analyzed using generalized ANOVA, where residual variances were estimated separately for each group, such that the homoscedasticity assumption must not be necessarily fulfilled. We used linear regression analyses stratified by groups to investigate associations between clinical parameters (BMI, fasting blood glucose (FBG), triglycerides (TG), serum glutamic-pyruvic transaminase (SGPT), nonesterified fatty acids (NEFA), visceral and subcutaneous fat, liver lipid content, EGP suppression during high-insulin clamp, M-value, $\dot{V}$O_2_ max) and mitochondrial capacity, mitochondrial protein levels, marker of oxidative stress and lipid metabolism obtained from the three groups (T2D, IR-NDM, and IS-NDM) after detraining. All analysis were adjusted for age and BMI at baseline. P-values from two-sided tests less than or equal to 5% were considered to indicate significant differences. All analyses were performed using SAS version 9.4 (RRID: SCR_008567; SAS Institute, Cary, NC, USA).

### Role of funders

The funding source of this study played no role in the study design, data collection, analysis, interpretation, writing, or editing of the manuscript.

## Results

### Effects of detraining on anthropometric and laboratory characteristics

Out of the 49 participants of the HIIT study, 20 T2D and 22 individuals without diabetes (NDM) also participated in the detraining study (Supplementary Fig. S1). Data of anthropometric and laboratory characteristics for all HIIT participants at baseline and changes after 12-week HIIT compared to baseline have been reported before.^24^

Supplementary Table S4 reports the data for the T2D and NDM groups of the detraining study. At baseline, T2D had higher visceral and subcutaneous fat content, HbA1c, SGPT, FBG, fasting insulin (FINS), 2-h and 3-h PBG than NDM. In T2D, the HIIT-induced improvements of SGPT, NEFA, TG, high-density-lipoprotein cholesterol (HDL-C) and FBG^24^ persisted after detraining, while 2-h PBG after OGTT decreased after detraining as compared to HIIT. In NDM, only low-density-lipoprotein cholesterol (LDL-C), liver lipid content and $\dot{V}$O_2_ max were improved, whereas 2-h PBG was increased after HIIT. After detraining, we observed reduction of HbA1c, NEFA, FBG, 3-h PBG and sustained improvement of $\dot{V}$O_2_ max in the NDM group.

Among NDM, we separated IS-NDM from IR-NDM, using a cutoff M-value of 5.5 mg × g^−1^ × min^−1^ (high-insulin clamp conditions at baseline) for insulin resistance.^27^ In IR-NDM, the HIIT-induced reduction of FBG was sustained after detraining, while in IS-NDM only FINS was reduced after detraining (Table 1).

**Table 1:** Anthropometric and metabolic parameters at baseline, after 12 weeks of high-intensity interval training (12-week) and after 4 weeks of detraining in individuals with type 2 diabetes (T2D), insulin-resistant (IR-NDM) and insulin-sensitive (IS-NDM) glucose-tolerant humans.

Parameter	T2D			IR-NDM			IS-NDM		
	Baseline	12-week	Detraining	Baseline	12-week	Detraining	Baseline	12-week	Detraining
Age (years)	57 ± 1 (n = 20)			56 ± 1 (n = 10)			58 ± 1 (n = 12)		
BMI (kg/m^2^)	31.2 ± 0.6 (n = 20)	30.8 ± 0.6 (n = 20)	30.8 ± 0.7 (n = 20)	32.2 ± 0.7 (n = 10)	32.1 ± 0.8 (n = 10)	32.0 ± 0.7 (n = 10)	29.3 ± 0.6 (n = 12)	29.1 ± 0.4 (n = 12)	29.2 ± 0.5 (n = 12)
WBF (kg)	27 (22, 32) (n = 17)	26 (21, 33) (n = 17)	25 (21, 30) (n = 16)	29 (25, 35) (n = 10)	28 (25, 33) (n = 9)	30 (28, 32) (n = 7)	23 (21, 29) (n = 10)	22 (21, 27) (n = 8)	23 (20, 26) (n = 10)
VF (kg)	6 (4, 7) (n = 17)	6 (4, 7)∗ (n = 17)	6 (4, 7) (n = 16)	5 (4, 6) (n = 10)	6 (5, 6) (n = 9)	6 (3, 6) (n = 7)	4 (3, 5) (n = 10)	4 (3, 5) (n = 8)	4 (3, 5) (n = 10)
SF (kg)	19 (17, 25) (n = 17)	20 (17, 26) (n = 17)	20 (16, 24) (n = 16)	24 (20, 29) (n = 10)	22 (20, 27) (n = 9)	25 (22, 29) (n = 7)	19 (16, 24)^†^ (n = 10)	18 (16, 23) (n = 8)	19 (17, 22) (n = 10)
HbA1c (%)	6.8 (6.4, 7.9) (n = 20)	6.8 (6.2, 7.2) (n = 20)	6.7 (6.2, 7.2) (n = 20)	5.3 (5.1, 5.7)^†^^††^ (n = 10)	5.2 (5.1, 5.8) (n = 10)	5.3 (5.0, 5.6) (n = 10)	5.4 (5.3, 5.6)^†††^ (n = 12)	5.5 (5.2, 5.6) (n = 12)	5.3 (5.2, 5.6) (n = 12)
SGPT (U/l)	41 (29, 62) (n = 20)	33 (25, 61) (n = 20)	29 (24, 47)∗∗∗ (n = 20)	30 (19, 38)^†^ (n = 9)	25 (18, 37) (n = 10)	21 (19, 29) (n = 10)	24 (21, 29)^††^ (n = 12)	24 (20, 29) (n = 12)	23 (19, 27) (n = 11)
NEFA (μmol/l)	518 (337, 669) (n = 19)	336 (275, 467)∗∗ (n = 19)	390 (291, 479)∗∗ (n = 19)	485 (323, 599) (n = 10)	408 (333, 532) (n = 10)	305 (260, 437)∗ (n = 10)	453 (239, 506) (n = 12)	369 (273, 543) (n = 12)	321 (189, 503) (n = 11)
TG (mg/dl)	152 (125, 231) (n = 20)	119 (90, 173)∗∗ (n = 20)	117 (105, 182)∗ (n = 20)	131 (99, 165) (n = 10)	133 (100, 148) (n = 9)	114 (95, 161) (n = 9)	121 (82, 149) (n = 12)	128 (81, 160) (n = 12)	120 (81, 172) (n = 12)
LDL-C (mg/dl)	132 (99, 171) (n = 20)	129 (98, 159) (n = 20)	126 (102, 152) (n = 20)	154 (137, 176)^†^ (n = 10)	143 (125, 166) (n = 10)	155 (122, 167) (n = 9)	158 (120, 166) (n = 12)	130 (118, 155)∗ (n = 12)	151 (123, 162) (n = 12)
HDL-C (mg/dl)	41 (36, 56) (n = 20)	51 (40, 58)∗∗∗ (n = 20)	48 (42, 59)∗∗ (n = 20)	53 (50, 63)^†^ (n = 10)	55 (50, 63) (n = 10)	55 (49, 67) (n = 9)	52 (47, 62)^†^ (n = 12)	50 (43, 54)∗ (n = 12)	52 (45, 59) (n = 12)
FBG (mg/dl)	140 (116, 187) (n = 20)	132 (107, 168)∗ (n = 20)	129 (105, 170)∗∗ (n = 20)	87 (80, 89)^†^^††^ (n = 10)	80 (77, 85)∗ (n = 10)	82 (73, 88)∗ (n = 10)	87 (83, 90)^†††^ (n = 12)	85 (84, 89) (n = 12)	85 (81, 89) (n = 12)
FINS (μU/ml)	14 (8, 25) (n = 20)	16 (11, 24) (n = 20)	14 (11, 20) (n = 17)	10 (8, 14) (n = 8)	8 (6, 13) (n = 10)	10 (7, 14) (n = 8)	8 (5, 10)^††^ (n = 12)	8 (6, 10) (n = 12)	8 (6, 11)∗ (n = 12)
2-h PBG (mg/dl)	220 (179, 310) (n = 17)	243 (192, 298) (n = 18)	213 (135, 273)^#^ (n = 16)	85 (76, 95)^†^^††^ (n = 8)	88 (69, 110) (n = 10)	88 (70, 102) (n = 9)	78 (55, 99)^†^^†^^†^ (n = 12)	93 (85, 110)∗ (n = 10)	81 (72, 94) (n = 8)
3-h PBG (mg/dl)	186 (101, 247) (n = 17)	160 (114, 245) (n = 18)	158 (118, 225) (n = 17)	60 (55, 66)^†^^††^ (n = 8)	65 (61, 78) (n = 10)	63 (56, 65) (n = 9)	61 (53, 64)^†^^††^ (n = 12)	61 (50, 70) (n = 9)	54 (45, 67) (n = 8)

Abbreviations: BMI, body mass index; WBF, whole-body fat content; VF, visceral fat content; SF, subcutaneous fat content; HbA1c, hemoglobin A1c; SGPT, serum glutamic pyruvic transaminase; NEFA, non-esterified fatty acids; TG, triglycerides; LDL-C, low-density-lipoprotein cholesterol; HDL-C, high-density-lipoprotein cholesterol; FBG, fasting blood glucose; FINS, fasting insulin 2-h/3-h; PBG, plasma blood glucose at 2 and 3 h of OGTT.

Data are shown as mean ± SEM or median (q1, q3); ∗p < 0.05, ∗∗p < 0.01, ∗∗∗p < 0.001 vs baseline; ^#^p < 0.05 vs 12-week; ^†^p < 0.05, ^††^p < 0.01, ^†††^p < 0.001 vs T2D; linear mixed models for changes between the different time points (baseline, 12-week HIIT and detraining) and generalized ANOVA for differences between groups at baseline. All analyses are adjusted for age and BMI. Comparison of 12-week vs baseline data has been previously published.^24^

Finally, capillary glucose and plasma insulin levels during steady state of both stages of the clamp did not differ between time points in any group (glucose in mg/dl: T2D 89 ± 1 vs 90 ± 1 vs 90 ± 1; IR-NDM 89 ± 2 vs 89 ± 1 vs 90 ± 1; IS-NDM 89 ± 2 vs 90 ± 1 vs 90 ± 2. Insulin in μU/ml during high-insulin clamp: T2D 60 (45, 64) vs 59 (47, 67) vs 49 (43, 66); IR-NDM 48 (33, 68) vs 51 (46, 65) vs 49 (46, 59); IS-NDM 60 (45, 64) vs 59 (48, 64) vs 62 (53, 70). Insulin in μU/ml during low-insulin clamp: T2D 25 (23, 28) vs 25 (23, 33) vs 23 (21, 32); IR-NDM 20 (18, 33) vs 26 (20, 29) vs 22 (19, 30); IS-NDM 26 (21, 34) vs 26 (21, 29) vs 25 (20, 32)).

### Effects of detraining on insulin sensitivity

**Fig. 1:**
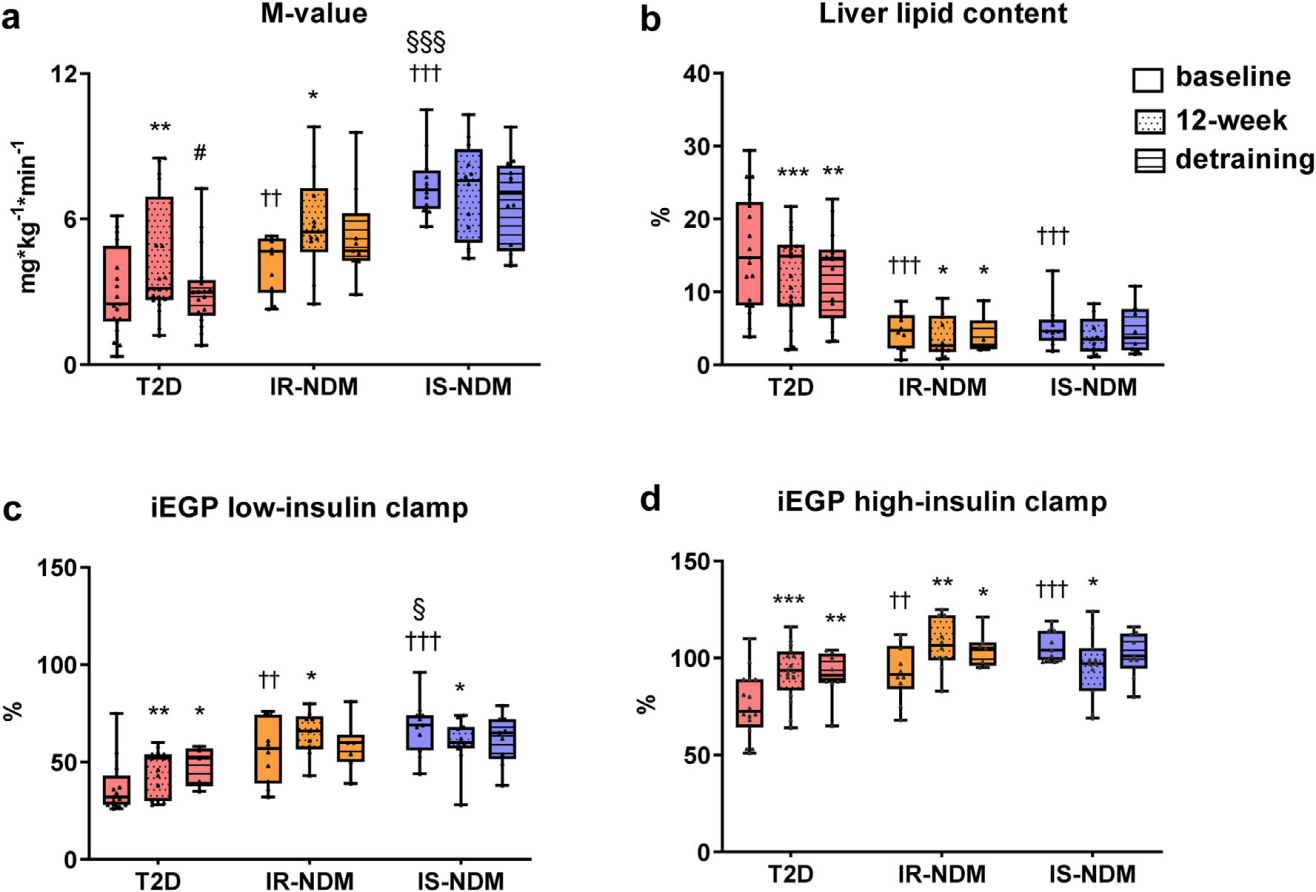
**Effects of detraining on whole-body and hepatic insulin sensitivity and liver lipid content in icinsulin-sensitive and insulin-resistant humans. (a)** Whole-body insulin sensitivity (M-value), **(b)** liver lipid content, **(c)** hepatic insulin sensitivity (iEGP) during low- and **(d)** high-insulin clamp, at baseline, after 12-week HIIT and after 4-week detraining in persons with T2D, IR-NDM and IS-NDM. ∗p < 0.05, ∗∗p < 0.01, and ∗∗∗p < 0.001 vs baseline; ^#^p < 0.05 vs 12-week; ^††^p < 0.01, ^†††^p < 0.001 vs T2D; ^§^p < 0.05, ^§§§^p < 0.001 vs IR-NDM; linear mixed models for changes between the different time points (baseline, 12-week HIIT and detraining) and generalized ANOVA for differences between groups at baseline. All analyses are adjusted for age and BMI. Comparison of 12-week vs baseline data has been previously published.^24^

As previously reported, the high-insulin clamp-derived M-value was lower in T2D and IR-NDM than IS-NDM at baseline.^24^ The HIIT-induced improvement in M-value observed in both insulin-resistant groups,^24^ decreased to baseline values in T2D after detraining, but remained unaltered in IR-NDM (Fig. 1a). Improvements in liver lipid content and iEGP were maintained after detraining in T2D and IR-NDM (Fig. 1b–d). M-value as well as liver lipid content and iEGP did not change in IS-NDM during the whole study (Fig. 1a–d).

### Effects of detraining on skeletal muscle mitochondrial function

**Fig. 2:**
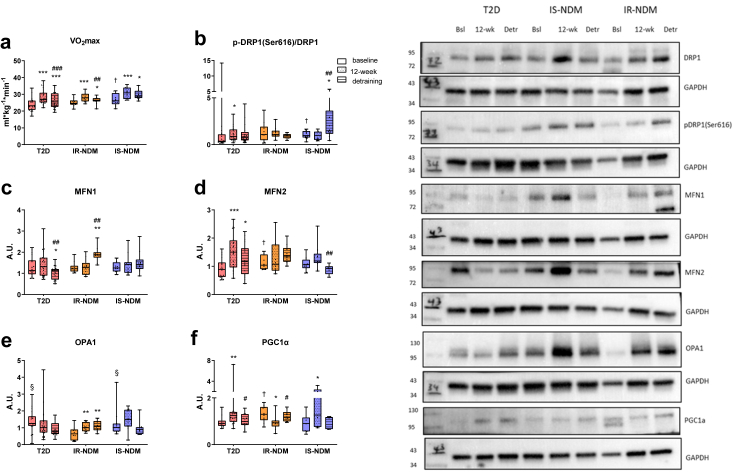
**Effects of detraining on mitochondrial dynamics in insulin-sensitive and insulin-resistant persons. (a)** Maximal oxygen uptake, and western blot analysis of **(b)** p-DRP1(Ser616)/DRP1, **(c)** MFN1, **(d)** MFN2, **(e)** OPA1, and **(f)** PGC1α at baseline, after 12-week HIIT and after 4-week detraining in T2D, IR-NDM and IS-NDM. Insets are representative Western blots for 1 participant for each time point. ∗p < 0.05, ∗∗p < 0.01, and ∗∗∗p < 0.001 vs baseline; ^#^p < 0.05, ^##^p < 0.01, ^###^p < 0.001 vs 12-week; ^†^p < 0.05 vs T2D; ^§^p < 0.05 vs IR-NDM; linear mixed models for changes between the different time points (baseline, 12-week HIIT and detraining) and generalized ANOVA for differences between groups at baseline. All analyses are adjusted for age and BMI.

After detraining, the HIIT-stimulated $\dot{V}$O_2_ max declined in T2D and IR-NDM, but remained higher compared to baseline in all groups (Fig. 2a). Conversely, muscle mitochondrial capacity remained similar compared to the 12-week HIIT values, except for mass-specific (corrected for tissue wet weight) leak respiration with electron input through complex I (CI; CI_L_), which returned to baseline levels in IS-NDM (Table 2).

**Table 2:** Mass-specific mitochondrial respiratory capacity in skeletal muscle of T2D, IR-NDM, and IS-NDM at baseline, after 12 weeks of HIIT (12-week) and 4 weeks of training pause (detraining).

Parameter	T2D			IR-NDM			IS-NDM		
	Baseline	12-week	Detraining	Baseline	12-week	Detraining	Baseline	12-week	Detraining
[CI]_L_	5.5 (4.2, 6.5) (n = 16)	7.4 (5.5, 8.3) (n = 17)	5.5 (3.9, 8.4) (n = 14)	9.1 (8.1, 11)^††^ (n = 8)	6.6 (5.4, 9.1) (n = 9)	8.3 (7.3, 10) (n = 8)	6.9 (5.6, 8.1) (n = 12)	10 (7.7, 12)∗∗ (n = 12)	6.1 (5.6, 7.2)^###^ (n = 11)
[CI]_P_	28 (20, 36) (n = 14)	27 (25, 46) (n = 17)	26 (22, 42) (n = 14)	38 (28, 47)^†^ (n = 8)	47 (28, 53) (n = 9)	37 (29, 46) (n = 8)	34 (23, 38) (n = 12)	52 (36, 57)∗∗∗ (n = 12)	43 (32, 50)∗ (n = 11)
[CI + II]_P_	50 (43, 56) (n = 16)	55 (42, 81) (n = 17)	45 (40, 68) (n = 15)	70 (48, 81) (n = 7)	71 (43, 91) (n = 8)	63 (47, 74) (n = 8)	56 (42, 60) (n = 12)	84 (60, 91)∗∗ (n = 12)	70 (61, 83)∗∗ (n = 10)

Abbreviations: [CI]L, leak respiration with electron input through complex I; [CI]P, oxidative phosphorylation (OXPHOS) capacity with electron input through CI; [CI + II]P, OXPHOS capacity with convergent electron input through CI and CII.

Oxygen flux rates corrected for tissue wet weight and are expressed as pmol O_2_ × mg wet weight^−1^ × s^−1^.

Data are shown as median (q1, q3); ∗p < 0.05, ∗∗p < 0.01, ∗∗∗p < 0.001 vs baseline; ^###^p < 0.001 vs 12-week; ^†^p < 0.05, ^††^p < 0.01 vs T2D; linear mixed models for changes between the different time points (baseline, 12-week HIIT and detraining) and generalized ANOVA for differences between groups at baseline. All analyses are adjusted for age and BMI. Comparison 12-week vs baseline has been previously published.^24^

As changes in physical activity and detraining could modulate oxidative capacity and insulin resistance via mitochondrial alterations, we assessed muscle mitochondrial dynamics and turnover.^4^ The mitochondrial fission from DRP1 (dynamin-related protein 1) activation, as calculated from the p-DRP1(Ser616)/total DRP1 protein ratio^3^ (Supplementary Fig. S3a and b), was significantly higher in T2D than in insulin-sensitive individuals at baseline (Fig. 2b). Of note, activated DRP1 was reduced after HIIT and remained unaltered after detraining in T2D, but increased in IS-NDM (Fig. 2b).

Conversely, markers of mitochondrial fusion increased after HIIT and detraining in T2D (MFN2, Mitofusin 2) and IR-NDM (Optic Atrophy 1, OPA1) but decreased in IS-NDM (MFN2). Of note, MFN1, essential for OPA1-driven fusion,^40^ was increased in IR-NDM but reduced in T2D after detraining (Fig. 2c–e). Furthermore, HIIT-induced changes in protein expression of PGC1α (Peroxisome Proliferator-activated Receptor gamma Coactivator 1 α), a key regulator of mitochondrial biogenesis,^4^ returned to baseline levels in T2D and IR-NDM after detraining (Fig. 2f), in accordance with a previous study.^19^

**Fig. 3:**
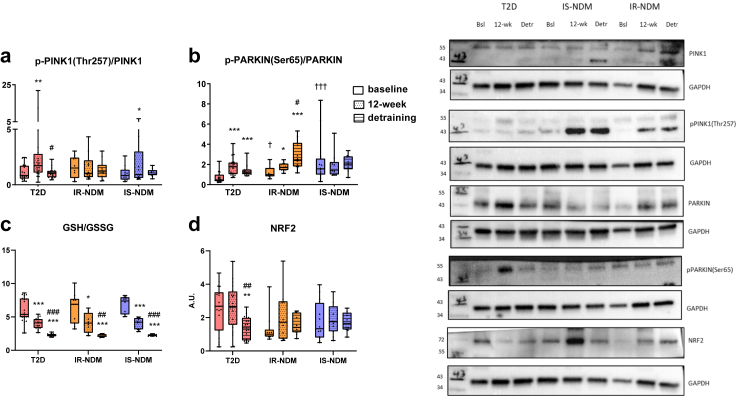
**Effects of detraining on mitophagy, cellular oxidative stress and antioxidant activity in insulin-sensitive and insulin-resistant humans.** Western blot analysis of **(a)** p-PINK1(Thr257)/PINK1 and **(b)** p-PARKIN(Ser65)/PARKIN, **(c)** GSH/GSSG ratio and **(d)** protein levels of NRF2, at baseline, after 12-week HIIT and after 4-week detraining in the skeletal muscle of T2D, IR-NDM and IS-NDM. Insets are representative Western blots for 1 participant for each time point. ∗p < 0.05, ∗∗p < 0.01, and ∗∗∗p < 0.001 vs baseline; ^#^p < 0.05, ^##^p < 0.01, ^###^p < 0.001 vs 12-week; ^†^p < 0.05, ^†††^p < 0.001 vs T2D; linear mixed models for changes between the different time points (baseline, 12-week HIIT and detraining) and generalized ANOVA for differences between groups at baseline. All analyses are adjusted for age and BMI. Comparison of 12-week vs baseline data for data in c and d has been previously published.^24^

Activated PINK1 (PTEN Induced Kinase 1; p-PINK1(Thr257)/total PINK1 protein ratio) (Supplementary Fig. S3c and d), marker of mitophagy,^3^ increased after HIIT in T2D and IS-NDM (Fig. 3a), whereas activation of PARKIN (p-PARKIN(Ser65)/total PARKIN ratio) (Supplementary Fig. S3e and f), also marker of mitophagy,^3^ was observed after HIIT and after detraining in both insulin-resistant groups (Fig. 3b).

In addition, we assessed muscle oxidative stress by different independent measures. After detraining, the reduced-to-oxidized glutathione (GSH/GSSG) ratio, a biomarker of tissue redox state,^41^ was decreased in comparison to baseline and HIIT in all groups (Fig. 3c), due to decreased GSH and increased GSSG after detraining (Supplementary Fig. S4). Finally, antioxidant protein nuclear factor-like 2 (NRF2) was reduced in T2D after detraining (Fig. 3d).

### Effects of detraining on skeletal muscle lipid metabolism

**Fig. 4:**
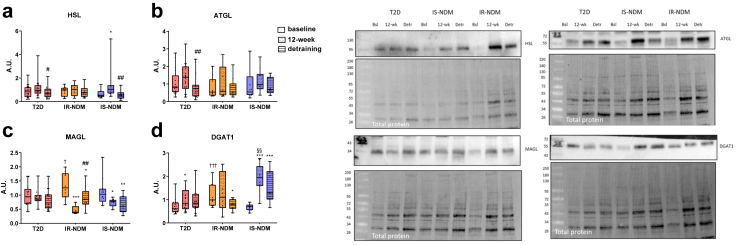
**Effects of detraining on skeletal muscle lipid metabolism.** Western blot analysis of **(a)** HSL, **(b)** ATGL, **(c)** MAGL, and **(d)** DGAT1, at baseline, after 12-week HIIT and after 4-week detraining in the skeletal muscle of T2D, IR-NDM and IS-NDM. Insets are representative Western blots for 1 participant for each time point. ∗p < 0.05, ∗∗p < 0.01, and ∗∗∗p < 0.001 vs baseline; ^#^p < 0.05, ^##^p < 0.01 vs 12-week; ^†^p < 0.05, ^†††^p < 0.001 vs T2D; ^§§^p < 0.01 vs IR-NDM; linear mixed models for changes between the different time points (baseline, 12-week HIIT and detraining) and generalized ANOVA for differences between groups at baseline. All analyses are adjusted for age and BMI.

The protein levels of HSL increased after HIIT in IS-NDM and decreased after detraining in T2D and IS-NDM (Fig. 4a). Similarly, the levels of ATGL decrease after detraining in T2D (Fig. 4b). Conversely, the levels of the monoacyl glycerol lipase (MAGL) were reduced after HIIT and stay unchanged after detraining in both glucose-tolerant groups (Fig. 4c). Finally, the protein levels of DGAT1 were increased after HIIT and remained unchanged after detraining in T2D and IS-NDM, whereas were reduced after detraining in IR-NDM (Fig. 4d).

### Association of clinical parameters with mitochondrial function and lipid metabolism at detraining

**Fig. 5:**
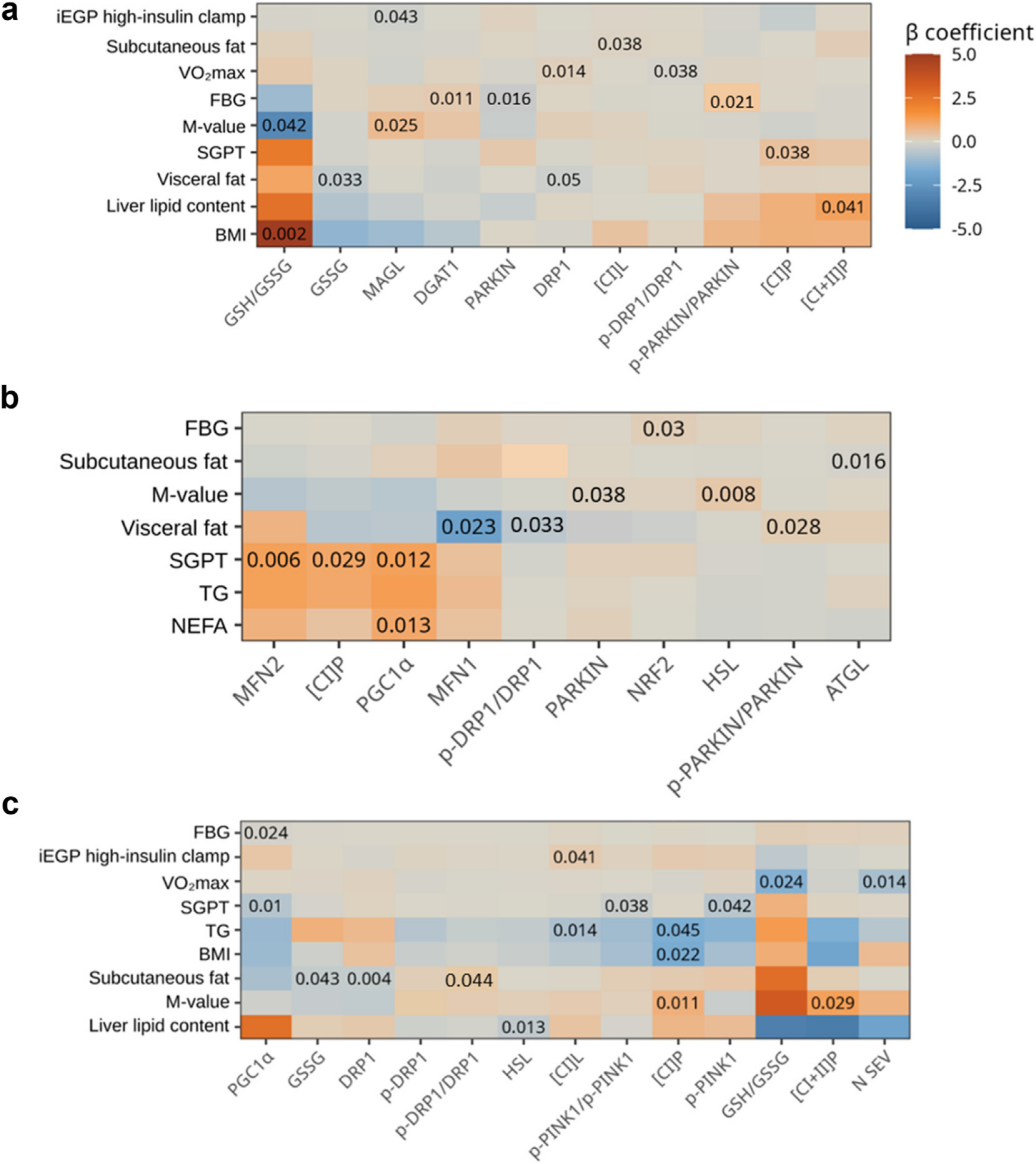
**Correlation of markers of energy metabolism and SEV number with clinical features observed after detraining.** Heatmaps depicting the correlation between anthropometric and metabolic parameters and mitochondrial capacity, marker of oxidative stress, protein levels detected by western blotting as well as number of SEV in **(a)** T2D, **(b)** IR-NDM, and **(c)** IS-NDM. Data are shown after adjustments for age and BMI. Red color denotes positive and blue color denotes negative correlation. Abbreviations: FBG, fasting blood glucose; SGPT, serum glutamic pyruvic transaminase; NEFA, non-esterified fatty acids; TG, triglycerides; M-value, whole body insulin sensitivity; BMI, body mass index; EGP, hepatic (iEGP) insulin sensitivity during high-insulin clamp.

Fig. 5 and Supplementary Table S5 show the associations between clinical parameters and mitochondrial capacity, mitochondrial protein levels, marker of oxidative stress and lipid metabolism. In T2D, M-value correlated negatively with the GSH/GSSG ratio (β = −3.003, p = 0.042) and FBG correlated positively with activated PARKIN (β = 0.420, p = 0.021). In IR-NDM, M-value associated positively with HSL (β = 0.350, p = 0.008), whereas in IS-NDM it associated positively with O_2_ fluxes (CI_P_: β = 0.904, p = 0.011; CI + II_P_: β = 1.262, p = 0.029).

### Effects of detraining on circulating SEV

**Fig. 6:**
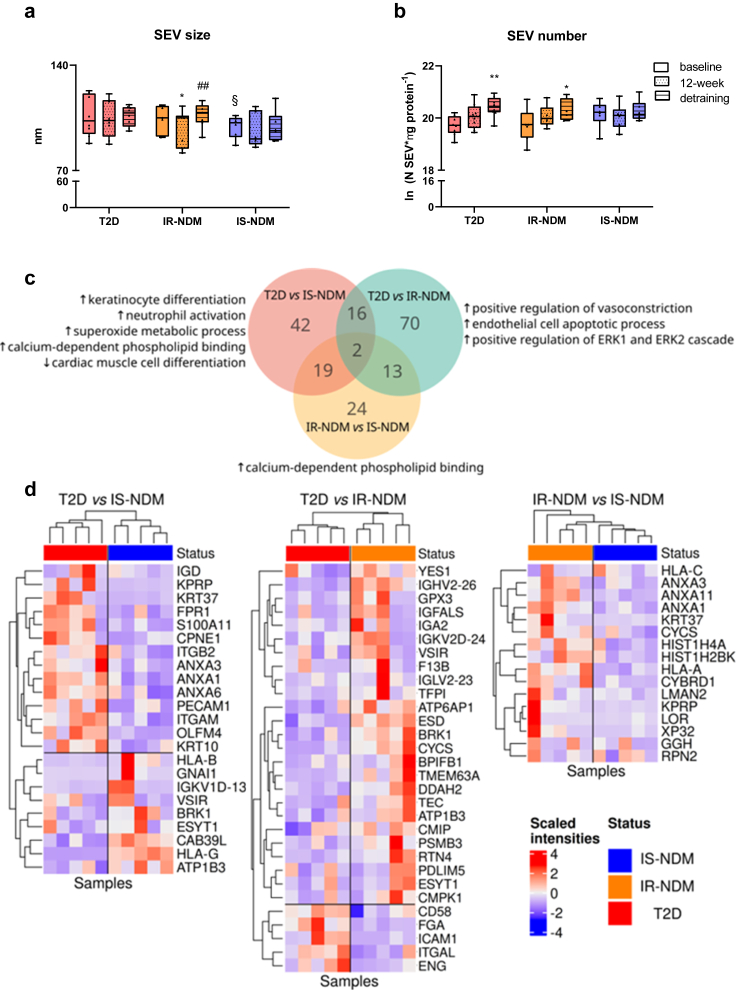
**Effects of detraining on HIIT-induced release of SEV and their contents in insulin-sensitive and insulin-resistant humans. (a)** SEV size and **(b)** SEV number normalized to μg of proteins at baseline, after 12-week HIIT and after 4-week detraining in insulin-resistant (T2D, n = 8; IR-NDM, n = 7) and insulin-sensitive (IS-NDM, n = 6) individuals. ∗p < 0.05, ∗∗p < 0.01 vs baseline; ^##^p < 0.01 vs 12-week; ^§^p < 0.05 vs IR-NDM; linear mixed models for changes between the different time points (baseline, 12-week HIIT and detraining) and generalized ANOVA for differences between groups at baseline. All analyses are adjusted for age and BMI. Comparison of 12-week vs baseline data has been previously published^24^; **(c)** Overlap of proteins differentially expressed between groups after detraining with GO terms of the differentially abundant proteins. The direction of the arrows indicates whether the proteins were up- or down-regulated in within each comparison; **(d)** Heatmaps of differentially abundant proteins (absolute average log2 ratio ≥0.58 and q-value ≤ 0.01) in within each comparison (T2D vs IS-NDM, T2D vs IR-NDM, IR-NDM vs IS-NDM).

The 12-week HIIT program triggered the release of SEV into the circulation in T2D and IR-NDM, but not IS-NDM.^24^ Here, we report size and number of circulating SEV upon detraining in the same subgroups of T2D (n = 8), IR-NDM (n = 7), and IS-NDM (n = 6). Average size of SEV was unchanged over the time, except for a slightly smaller size in IR-NDM after HIIT (Fig. 6a). After detraining, the number of SEV remained increased in insulin-resistant individuals (T2D, IR-NDM) and unchanged in IS-NDM (Fig. 6b).

Next, we characterized the protein cargo of SEV after detraining from T2D (n = 5), IR-NDM (n = 5), and IS-NDM (n = 5) individuals and identified a total of 1114 proteins (Supplementary Table S2). The GO-CC enrichment analysis performed on the SEV proteome confirmed an overrepresentation of proteins associated with extracellular exosomes (Supplementary Fig. S5a and Supplementary Table S2). The differential abundance analysis conducted on the protein dataset across the distinct groups (T2D, IR-NDM, IS-NDM) revealed 186 differentially abundant proteins (q-value ≤0.05, absolute log2 ratio >0.58; Fig. 6c and Supplementary Table S2). Notably, only 2 of the 186 proteins were different between all groups and expressed at the lowest levels in T2D (Supplementary Fig. S5b). We also found that 44% (81 of 186) of these proteins did not have a predicted secretory signal peptide (Supplementary Fig. S5c and Supplementary Table S2), implying that SEV might represent a new mechanism for the release of exerkines, independent of classic secretory pathways.

### Detraining differently affects SEV protein expression among groups

We subsequently subjected the 186 SEV proteins, which were differentially regulated between groups after detraining, to gene ontology enrichment analysis. We performed separate enrichment analysis for the proteins up- and down-regulated within each group comparison (T2D vs IS-NDM, T2D vs IR-NDM, IR-NDM vs IS-NDM).

SEV proteins upregulated in T2D vs IS-NDM individuals included among others peroxiredoxin 1 (PRDX1), formyl peptide receptor 1 (FPR1) and proteins involved in keratinocyte differentiation and in neutrophil activation, as revealed by GO-BP analysis (Fig. 6c and d and Supplementary Table S3).

Among the proteins upregulated in T2D vs IR-NDM, we found an enrichment of proteins involved in the extracellular signal-regulated kinase 1/2 (ERK1/2) cascade (Fig. 6c and Supplementary Table S3). Conversely, proteasome 20S subunit beta 3 (PSMB3) and glutathione peroxidase 3 (GPX3) were upregulated in IR-NDM in comparison to T2D (Fig. 6d and Supplementary Table S3). Besides GPX3, the expression of other proteins with peroxidase activity, prostaglandin-endoperoxide synthase 1 (PTGS1) and PRDX2, was higher in SEV from IR-NDM vs the other groups (Supplementary Table S2).

We further found that the expression of 162 SEV proteins differed between people with T2D and those without diabetes (IS-NDM and IR-NDM) (Supplementary Table S2). Of those, 13 proteins were also differentially regulated between T2D and NDM groups at baseline and after HIIT, comprising myeloperoxidase (MPO), which was higher in T2D than in both glucose-tolerant groups after detraining.

Importantly, the expression of 116 other SEV-derived proteins differed between the insulin-resistant (IR-NDM and T2D) and the insulin-sensitive group after detraining (Supplementary Table S2) and upregulated proteins were associated with the GO-MF term “calcium-dependent phospholipid binding”, which comprises among others Copine 1 (CPNE1) (Fig. 6c and Supplementary Table S3).

### Correlation of specific SEV proteins with insulin sensitivity and adipose tissue volume after detraining

**Fig. 7:**
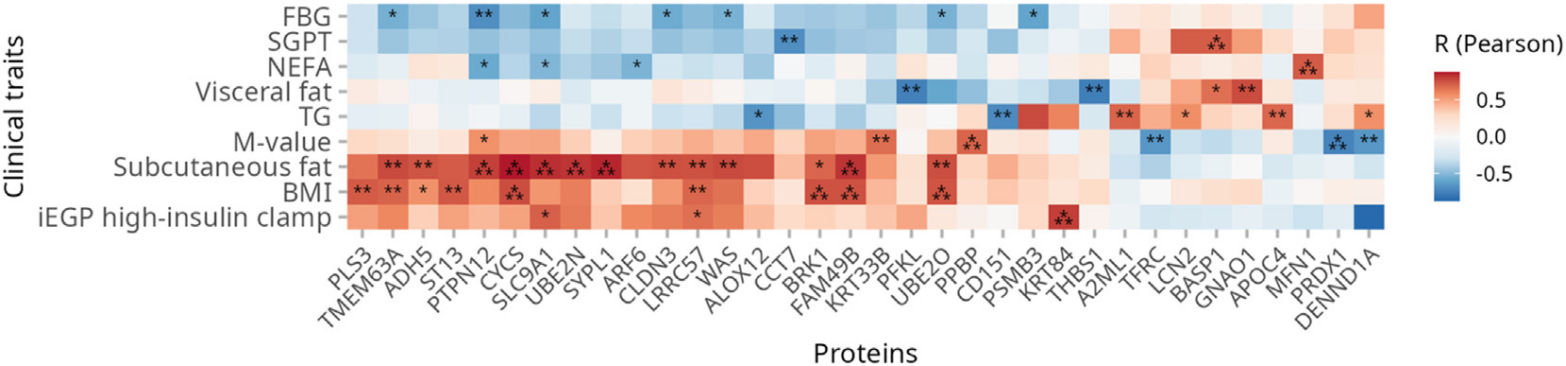
**Correlation of specific SEV proteins with clinical features measured after detraining.** Heatmap depicting the Pearson correlation between levels of SEV proteins and clinical parameters measured at detraining in all participants. Data are shown after adjustments for age and BMI. Red color denotes positive and blue color denotes negative correlation; each cell with an asterisk refers to a significant Pearson correlation (∗p < 0.05, ∗∗p < 0.01, ∗∗∗p < 0.001). Cells without asterisk indicate that the correlations did not reach the statistical significance. Abbreviations: FBG, fasting blood glucose; SGPT, serum glutamic pyruvic transaminase; NEFA, non-esterified fatty acids; TG, triglycerides; M-value, whole body insulin sensitivity; BMI, body mass index; EGP, hepatic (iEGP) insulin sensitivity during high-insulin clamp.

Correlation analysis revealed several statistically significant associations of SEV proteins with clinical parameters. Across all groups, transferrin receptor (TFRC) correlated negatively (R = −0.657, p = 0.008; R_S_ = −0.707, p = 0.003) with M-value, arachidonate 12-lipoxygenase (ALOX12) correlated negatively with TG (R = −0.671, p = 0.012; R_S_ = −0.641, p = 0.018), and thrombospondin 1 (THBS1) negatively with visceral fat volume (R = −0.763, p = 0.006; R_S_ = −0.7, p = 0.016) (Fig. 7, Supplementary Fig. S6, and Supplementary Table S6).

## Discussion

This study shows that a 4-week detraining period decreases—without reversing—the HIIT-induced increase in whole-body oxidative metabolism in insulin-resistant individuals (T2D, IR-NDM) and diminishes the HIIT-induced increase in whole-body insulin sensitivity in T2D. However, it does not affect improvements in hepatic insulin sensitivity, hepatic lipid content, and glycemia in insulin-resistant individuals. Furthermore, detraining maintains muscle mitochondrial fusion and mitophagy in insulin-resistant individuals but stimulates mitochondrial fission in the insulin-sensitive individuals. It also reduces the expression of muscle lipases in T2D (ATGL, HSL) and in glucose-tolerant individuals (MAGL in IR-NDM, HSL and MAGL in IS-NMD). Finally, training cessation neither affects the HIIT-triggered increase in number of SEV in insulin-resistant individuals nor the overexpression of antioxidant proteins (PRDX1, PRDX2, GPX3, PTGS1).

Of note, after detraining $\dot{V}$O_2_ max remained unchanged only in IS-NDM, suggesting that HIIT-stimulation of oxidative metabolism declines earlier in insulin-resistant persons, as previously described in people with impaired glucose tolerance.^18^ However, $\dot{V}$O_2_ max remained higher compared to baseline values in all groups in contrast to other previous studies examining detraining effects after aerobic exercising.^10^, ^11^, ^12^^,^^15^ These data highlight the superior sustainability of cardiometabolic benefits of HIIT during detraining.

Interestingly, HIIT-stimulated whole-body insulin sensitivity (M-value) decreased only in T2D in line with prior research indicating that increased whole-body insulin sensitivity—stimulated by other training modalities—persists primarily in lean individuals.^14^ Conversely, HIIT-induced reduction in hepatic insulin resistance (iEGP) and lipid content in T2D and IR-NDM persisted after 4-week detraining, possibly reflecting sustained lowering lipid flux to the liver and intrahepatic lipid mediators such as DAG or ceramides, known to inhibit hepatic insulin signaling.^9^ In the absence of liver biopsies, the decrease of circulating NEFA and adipose tissue volume may serve as surrogate of reduced lipid flux to and their accumulation in the liver. Also, TG and HDL-C remained improved after detraining in T2D as shown before in obese individuals.^42^ Surprisingly, detraining decreased iEGP in IS-NDM, in line with previous studies using postprandial indices.^43^^,^^44^ This could result from compensatory adjustment of hepatic metabolism to reduce hypoglycemia risk after training. Indeed, 2-h PG transiently increased during OGTT in IS-NDM, in line with a previous study,^45^ and returned to baseline values after detraining.

This study further elucidated possible mechanisms involved in the sustainability of improved whole-body insulin sensitivity, such as mitochondrial respiration, dynamics, oxidative stress and lipid metabolism in skeletal muscle.^9^^,^^46^

Mitochondrial respiratory capacity was unaltered in insulin-resistant individuals, whereas HIIT-induced CI_L_ declined in IS-NDM after detraining, confirming the exercise-stimulated rise in leak respiration^47^ and decline after training.^48^ However, IS-NDM retained the HIIT-induced increase in OXPHOS capacity through CI and CII, which may help maintain whole-body insulin sensitivity, as supported by the positive association with M-value in our regression analysis.

Previous studies indicate that exposure to nutrient excess, such as in obesity and T2D, promotes mitochondrial fission resulting in smaller muscle mitochondria.^4^ In line, we found that T2D show higher activated DRP1 than IS-NDM at baseline. Of note, HIIT reduced activated DRP1 in T2D and increased the fusion proteins MFN2 and OPA1 in both insulin-resistant groups. One may speculate that exercise training improves the ratio of fusion to fission proteins,^49^ which could contribute to improvement of insulin sensitivity after HIIT in both insulin-resistant participants and to its sustainability after detraining at least in IR-NDM. Similar to a previous study showing that physically active lifestyle promotes mitochondrial quality control by increasing fission and mitophagy,^50^ we also observed increased mitophagy (p-PINK1 and p-PARKIN) in T2D after HIIT, which might help to maintain mitochondrial respiratory function and insulin sensitivity.^51^ Of note, levels of p-PARKIN but not p-PINK1 were increased after detraining in both insulin-resistant groups, suggesting that PINK1 might not be critical for exercise-induced muscle mitophagy.^52^ Also, the reduced GSH/GSSG ratio after HIIT and detraining in all groups, suggests that exhaustive exercise may cause oxidative stress and damage mitochondria, resulting in enhanced mitophagy and reduced antioxidant activity (NRF2), as observed in T2D.

Another possible mechanism underlying exercise training-improved insulin sensitivity is by restoring lipase expression/activity and reducing lipid content in skeletal muscle.^53^ Similar to previous studies,^21^^,^^54^ ATGL protein levels were unaltered after HIIT in all participants, whereas HSL protein levels were increased after HIIT in IS-NDM, suggesting that exercise might contribute to insulin sensitivity via upregulation of HSL, which is responsible for DAG hydrolysis.^55^ Interestingly, the levels of both lipases were reduced after detraining in T2D and IS-NDM, which might in part explain the reversal of insulin sensitivity in T2D. Finally, the protein content of the lipase MAGL, which degrades monoacylglycerol to release fatty acids, was reduced after HIIT and detraining in both glucose-tolerant groups, supporting the hypothesis that monoacylglycerol accumulation attenuates insulin resistance.^56^ Additionally, this study shows that DGAT1 levels were increased after HIIT in T2D and IS-NDM, as previously shown in sedentary individuals after one bout of exercise.^57^ In conclusion, alterations in skeletal muscle lipolysis and lipid synthesis during exercise imply improved lipid handling possibly mediating improved skeletal muscle insulin sensitivity.

The regression analyses further pointed to various myocellular pathways of insulin sensitivity involved in metabolic outcomes at detraining. Notably, the inverse relationship of GSH/GSSG ratio and M-value, along with the positive correlation between activated PARKIN and FBG, support the hypothesis that oxidative stress-induced mitophagy may exacerbate insulin resistance in T2D.^46^ The positive association of M-value with HSL in IR-NDM support the hypothesis that the downregulation of HSL might lead to the accumulation of DAG and the development of insulin resistance.^58^ However, more studies are needed to confirm these findings and explore their clinical relevance.

Alternative mechanism operating during adaption to exercising could be the release of SEV, as we have previously proposed that increased SEV and their cargo may mediate changes in insulin sensitivity after HIIT.^24^ Interestingly, our current analysis shows that the number of SEV remained higher after detraining than before training in insulin-resistant individuals. Of note, the SEV proteome of insulin-resistant individuals displayed an upregulation of CPNE1, which is elevated in muscle of sarcopenic mice and reduces mitochondrial fusion and fission as well as exercise capacity.^59^ One can speculate that elevation of CPNE1 in SEV contributes to the reduction of whole-body oxidative capacity in the insulin-resistant humans after detraining.

Functional analysis of SEV proteins upregulated in T2D as compared to IS-NDM revealed an upregulation of FPR1, which might help explain the reduction of their insulin sensitivity after detraining, as the expression of this protein has been related to impaired glucose homeostasis in obese mice.^60^ Among the proteins upregulated in T2D as compared to IR-NDM, MPO and caspase recruitment domain 9 (CARD9), which play a role in inflammation and oxidative stress,^61^^,^^62^ may contribute to low-grade inflammation and the decline in insulin sensitivity of T2D after detraining. In line with this, the lower levels of GPX3, the major circulating antioxidant protein, in SEV of T2D compared to IR-NDM could further contribute to the reversal of improved insulin sensitivity in T2D after detraining. On the other hand, overexpression of proteins with peroxidase activity (PRDX2, GPX3, and PTGS1) in SEV from IR-NDM compared to the other groups (T2D and IS-NDM) suggests sustained protection from oxidative damage transported via SEV, which is in line with observations immediately after HIIT in this group.^24^ Finally, SEV from IR-NDM showed higher expression of the proteasome subunit PSMB3, which has been associated with improved muscle insulin sensitivity^63^ and could therefore also complement the mechanisms underlying the sustained improvement of insulin resistance in the IR-NDM group.

Correlation analysis suggested that several SEV proteins might be responsible for clinical outcomes at detraining. We found a negative correlation of TFRC with M-value and THBS1 with visceral fat, in line with previous studies.^64^, ^65^, ^66^ Moreover, the negative correlation between ALOX12 and TG suggests a potential role for ALOX12 in regulating lipid accumulation and distribution in T2D and obesity.^67^

Some limitations of this study have to be considered. First, this study was a monocentric study without population-based recruitment so that this cohort cannot be regarded as demographically representative. Specifically, only male participants were selected according to specific inclusion and exclusion criteria so that the findings cannot be generalized, e.g., because females might exhibit differences in muscle mass, fiber type composition and exercise response.^68^ Also, the study included only obese/overweight individuals to exclude the impact of different baseline body mass so that a general assessment of the effectiveness of HIIT and detraining cannot be made in the absence of a lean control group. Second, this study was powered for the primary outcome, i.e., changes in insulin sensitivity after HIIT training across all groups, but not for sub-analyses, e.g., according to insulin resistance or use of medication. Glucose-lowering medication was discontinued three days prior to the experiments in order to minimize any acute effects on examined parameters, which does not completely rule out effects of their long-term use. The small sample size precludes a subgroup analysis according to medication type. Third, despite the comprehensive assessment of mitochondrial oxidative capacity and quality control, direct morphological analysis of muscle mitochondria by transmission electron microscopy was not available. Fourth, it would have been valuable to directly compare the training and detraining SEV proteomic datasets, with baseline proteomic datasets. However, the datasets corresponding to detraining in comparison to baseline/HIIT were acquired using different mass spectrometers. As a consequence, notable variations arose within the datasets which could not be overcome by normalization, ultimately making a meaningful comparison unfeasible. As our study focused on characterizing the role of SEV in mediating the metabolic changes observed after detraining, we did not measure the presence of cell-specific markers in circulating SEV, which would be required to determine their tissue of origin. Fifth, although all participants underwent comprehensive metabolic phenotyping, MRI/MRS measurements and iEGP were measured in smaller subgroups of individuals at detraining due to practical issues or introduction of measurements later on in the study course. Finally, the HIIT training had been personally supervised and monitored, whereas the detraining period was monitored by physical exercise questionnaires, but without accelerometer monitoring.

In conclusion, our findings confirm that HIIT-induced improvements in whole-body oxidative metabolism and insulin sensitivity are not generally preserved after 4 weeks of detraining, but the reduction in hepatic lipid content and insulin sensitivity is sustained in insulin-resistant humans, and is reflected by improved glycemia. Greater mitochondrial turnover and downregulation of the lipase MAGL may contribute to the sustained improvement of whole-body insulin sensitivity in IR-NDM, whereas the downregulation of lipolytic enzymes in skeletal muscle of T2D may contribute to their decline of whole-body insulin sensitivity upon detraining. Finally, the metabolic changes observed after detraining may be attributed at least in part to HIIT-stimulated SEV release and SEV protein cargo, supporting a role of exosomes as mediators of exercise-induced inter-organ crosstalk.

## Contributors

MR initiated the investigation, secured funding for this study by the Ministry of Culture and Science of the State of Northrhine-Westphalia (MKW NRW), the German Federal Ministry of Health (BMG) as well as by a grant of the Federal Ministry for Research (BMBF) to the German Center for Diabetes Research (DZD e. V.), designed and led the clinical experiments and wrote the manuscript. LM designed and carried out the molecular analysis and the SEV experiments and wrote the manuscript. MA conducted clinical experiments, analyzed data and wrote the manuscript. PL performed the bioinformatics analysis. SH, SL, and HAH performed the MS experiments and analyzed the data. NT analyzed the respirometry data. KS performed statistical analysis. SG and YK contributed to clinical experiments. ST supervised laboratory analysis. JS designed the study and supervised clinical experiments. All authors read, reviewed and approved the final version of the manuscript. MR is the guarantor of this work and, as such, had full access to all the data in the study. LM, MA, and MR verified the underlying data of this manuscript.

## Data sharing statement

All data are available in the main text or the supplementary materials. Moreover, mass spectrometry-based proteomics data of serum SEV have been deposited to the ProteomeXchange Consortium via the PRIDE partner repository^25^ with the dataset identifier PXD046654.

## Declaration of interests

MR is currently on scientific advisory boards of Boehringer Ingelheim, Lilly, Novo Nordisk and has received personal fees from Echosens, Novo Nordisk and Target RWE, honoraria for lectures from Astra-Zeneca, Boehringer-Ingelheim, Novo Nordisk, Kenes Group, Madrigal, MSD and investigator-initiated research support from Boehringer-Ingelheim and Novo Nordisk. The research of MR is supported by grants from the European Community (HORIZON-HLTH-2022-STAYHLTH-02-01: Panel A) to the INTERCEPT-T2D consortium and German Research Foundation (DFG, GRK2576 Vivid). All other authors declare no competing interests.

## Appendix ASupplementary data

Supplementary Figures S1–S6

Supplemental Western Blots File
