# Supplementary material for: The role of exosomes for sustained specific cardiorespiratory and metabolic improvements in males with type 2 diabetes after detraining
Source: eBioMedicine. 2024 Dec 2;110:105471. doi: 10.1016/j.ebiom.2024.105471 (PMC11652844; doi:10.1016/j.ebiom.2024.105471)
Supplement: Supplementary Figures S1–S6 [file mmc7.docx]

Supplemental information for

**The role of exosomes for sustained specific cardiorespiratory and metabolic improvements in males with type 2 diabetes after detraining**

Lucia Mastrototaro *et al.*

*Corresponding author. Email: michael.roden@ddz.de


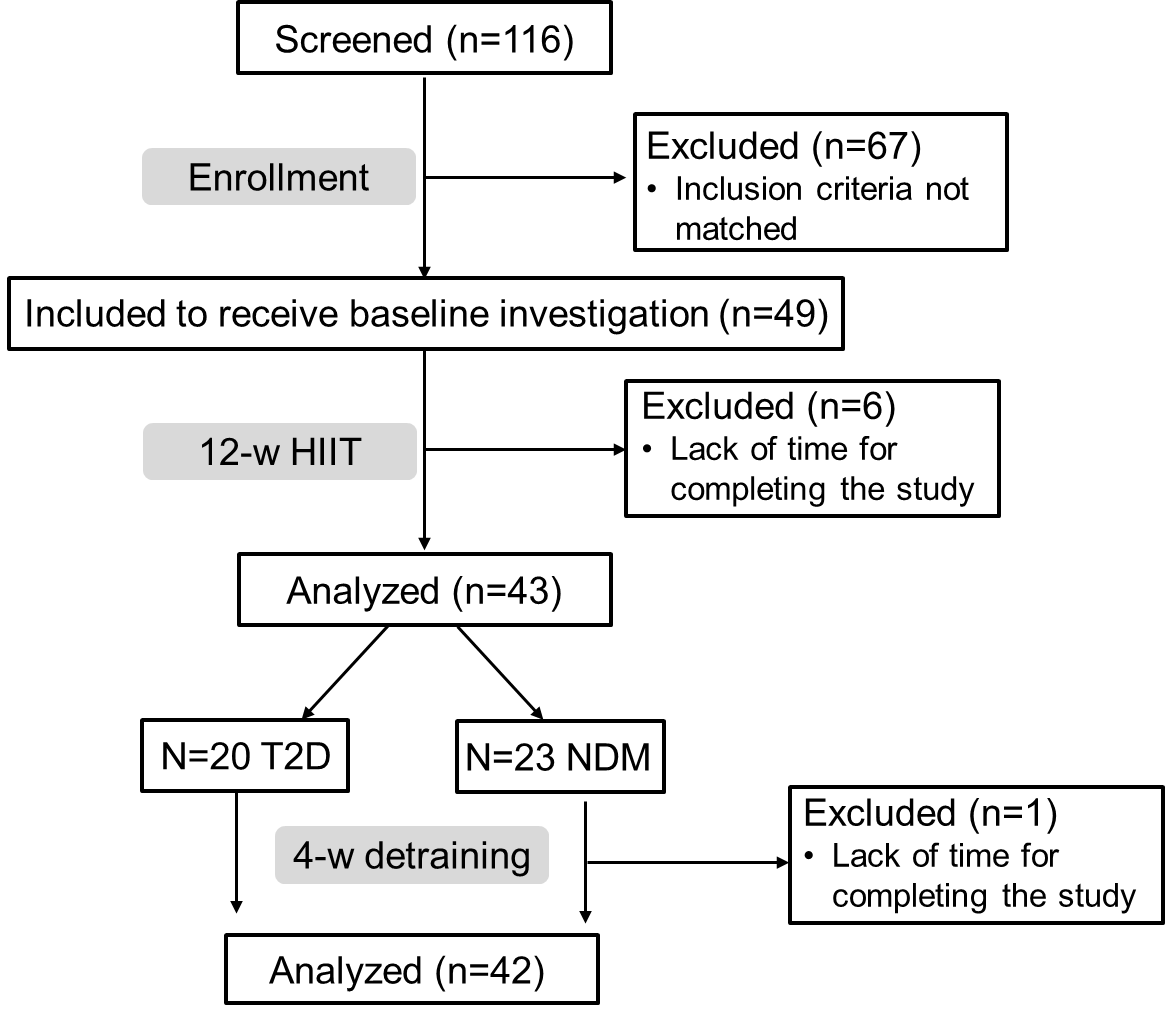


Fig. S1. Flow diagram of study design


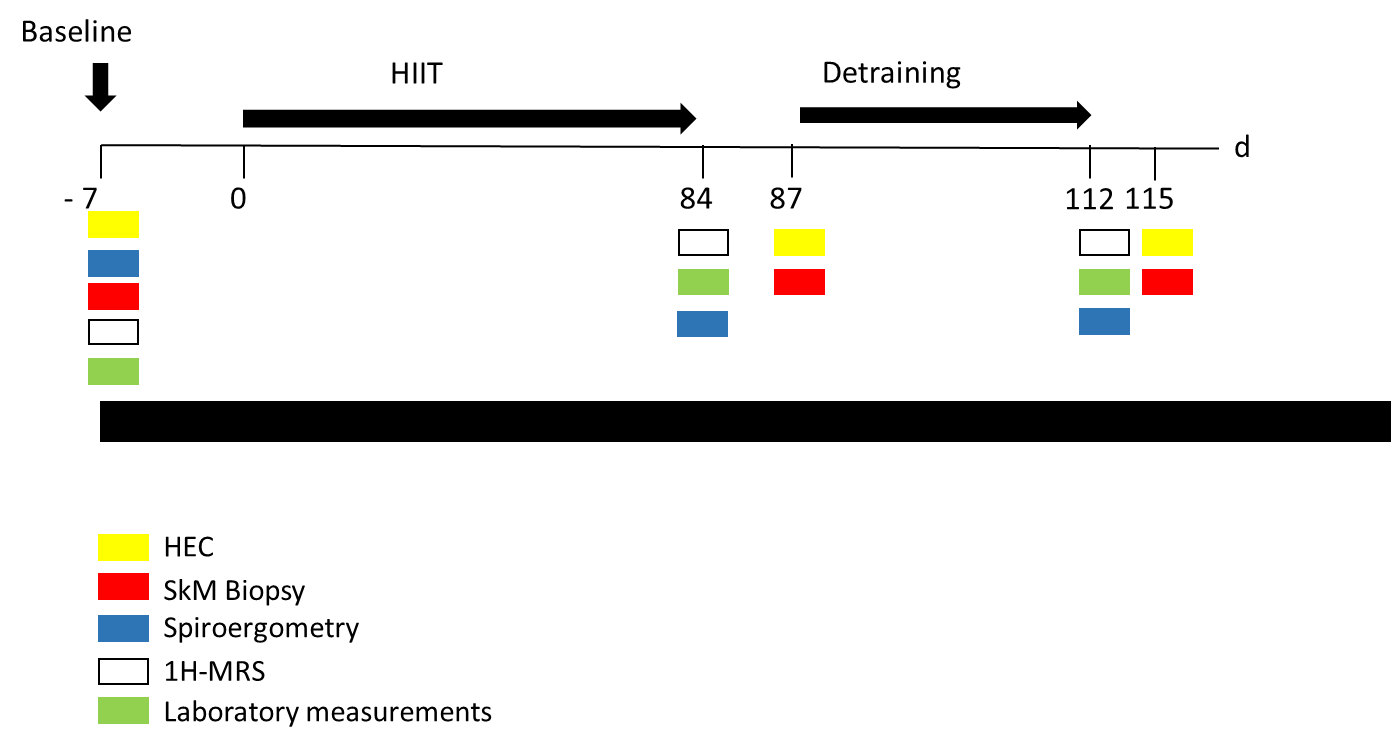


Fig. S2. Flow chart of the interventions conducted at baseline, after 12-week HIIT and 4-week detraining

**Fig. S3**. **Effects of detraining on mitochondrial dynamics and turnover in skeletal muscle of insulin resistant and insulin sensitive individuals**. Western blot analysis of **(a)** DRP1, **(b)** phospho-DRP1(Ser616), **(c)** PINK, **(d)** phospho-PINK1(Thr257), **(e)** PARKIN, **(f)** phospho-PARKIN(Ser65) at baseline, after 12-week HIIT and after 4-week detraining in persons with T2D, IR-NDM and IS-NDM. *p<0.05, **p<0.01 and ***p<0.001 vs baseline; ^#^p<0.05, ^##^p<0.01, ^###^p<0.001 vs 12-week; †p<0.05, ††p<0.01, †††p<0.001 vs T2D; §p<0.05 vs IR-NDM; linear mixed models for changes between the different time points (baseline, 12-week HIIT and detraining); generalized ANOVA for differences between groups at baseline.

Fig. S4. Effect of detraining on muscle oxidative stress. Concentration of (a) total, (b) free (GSH) and (c) oxidized (GSSG) glutathione in skeletal muscle of T2D, IR-NDM and IS-NDM**p<0.01 and ***p<0.001 vs baseline; ^#^p<0.05, ^##^p<0.01, ^###^p<0.001 vs 12-week; linear mixed models for changes between the different time points (baseline, 12-week HIIT and detraining); generalized ANOVA for differences between groups at baseline. Comparison of 12-week vs baseline data has been previously published in *[24]*.


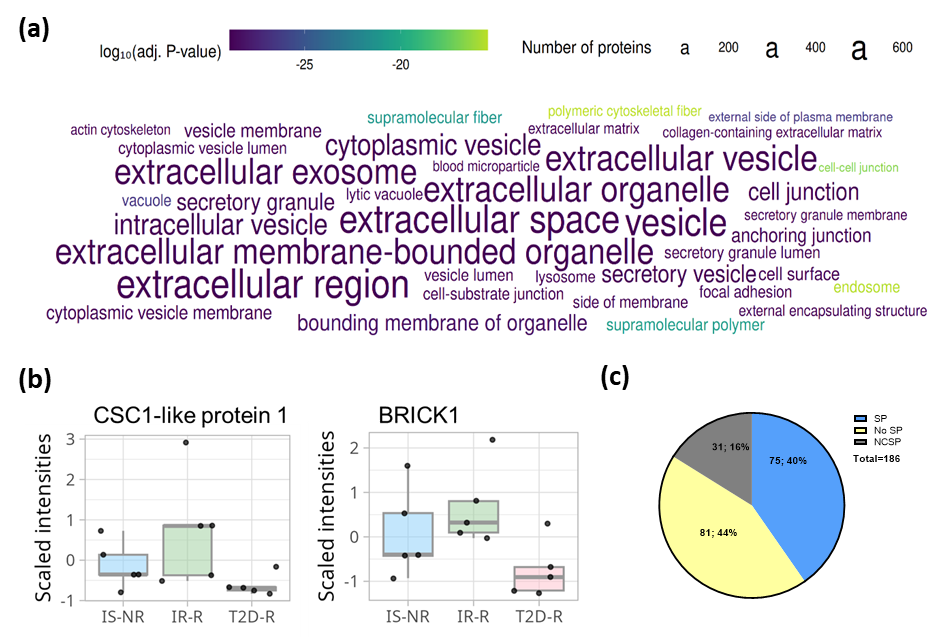


Fig. S5. Effects of detraining on the protein cargo of circulating SEV. GO-CC analysis of SEV proteins detected after detraining. GO terms are expressed as a word cloud with the size of the words indicating the number of proteins for each GO-CC term and the color indicating the B-H corrected p-value (a); boxplots of SEV proteins differentially expressed in all three groups (T2D, IR-NDM, IS-NDM) after detraining (b); amino acid sequences of the SEV proteins regulated after detraining (186 entries) were analyzed to predict the presence of a secretory signal peptide (SP) (c).


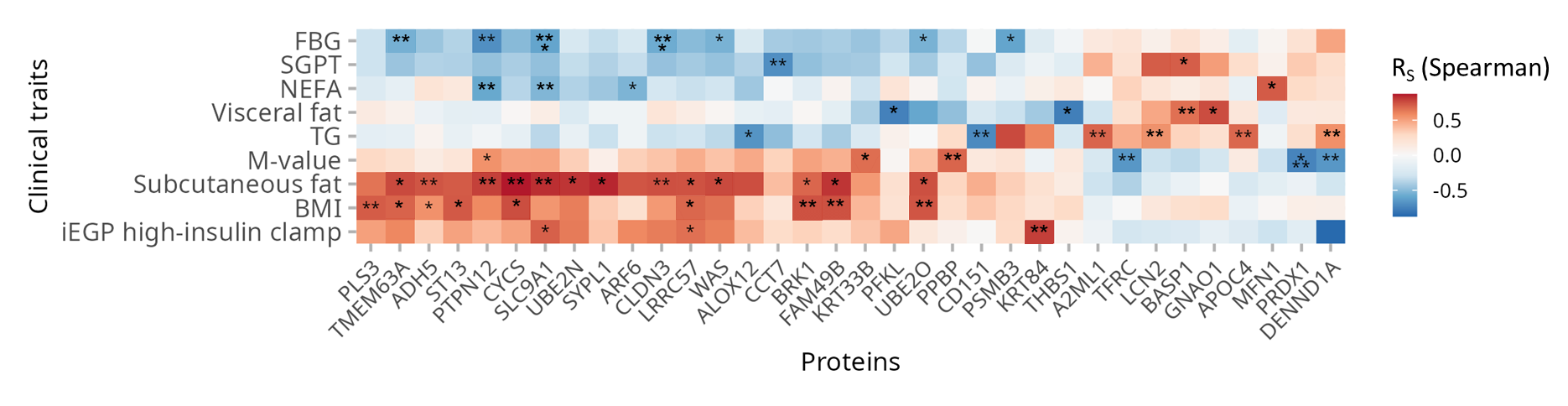


Fig. S6. Correlation of specific SEV proteins with metabolic parameters measured after detraining. Heatmap depicting the Spearman correlation between levels of SEV proteins and clinical parameters measured at detraining in all participants. Data are shown after adjustments for age and BMI. Red color denotes positive and blue color denotes negative correlation; each cell with an asterisk refers to a significant Spearman correlation (*p<0.05, **p<0.01, ***p<0.001). Cells without asterisk indicate that the correlations did not reach the statistical significance. Abbreviations: FBG, fasting blood glucose; SGPT, serum glutamic pyruvic transaminase; NEFA, non-esterified fatty acids; TG, triglycerides; M-value, whole body insulin sensitivity; BMI, body mass index; EGP, hepatic (iEGP) insulin sensitivity during high-insulin clamp.
