# Supplementary material for: The role of exosomes for sustained specific cardiorespiratory and metabolic improvements in males with type 2 diabetes after detraining
Source: eBioMedicine. 2024 Dec 2;110:105471. doi: 10.1016/j.ebiom.2024.105471 (PMC11652844; doi:10.1016/j.ebiom.2024.105471)
Supplement: Supplemental Western Blots File [file mmc8.pptx]

## Slide 1
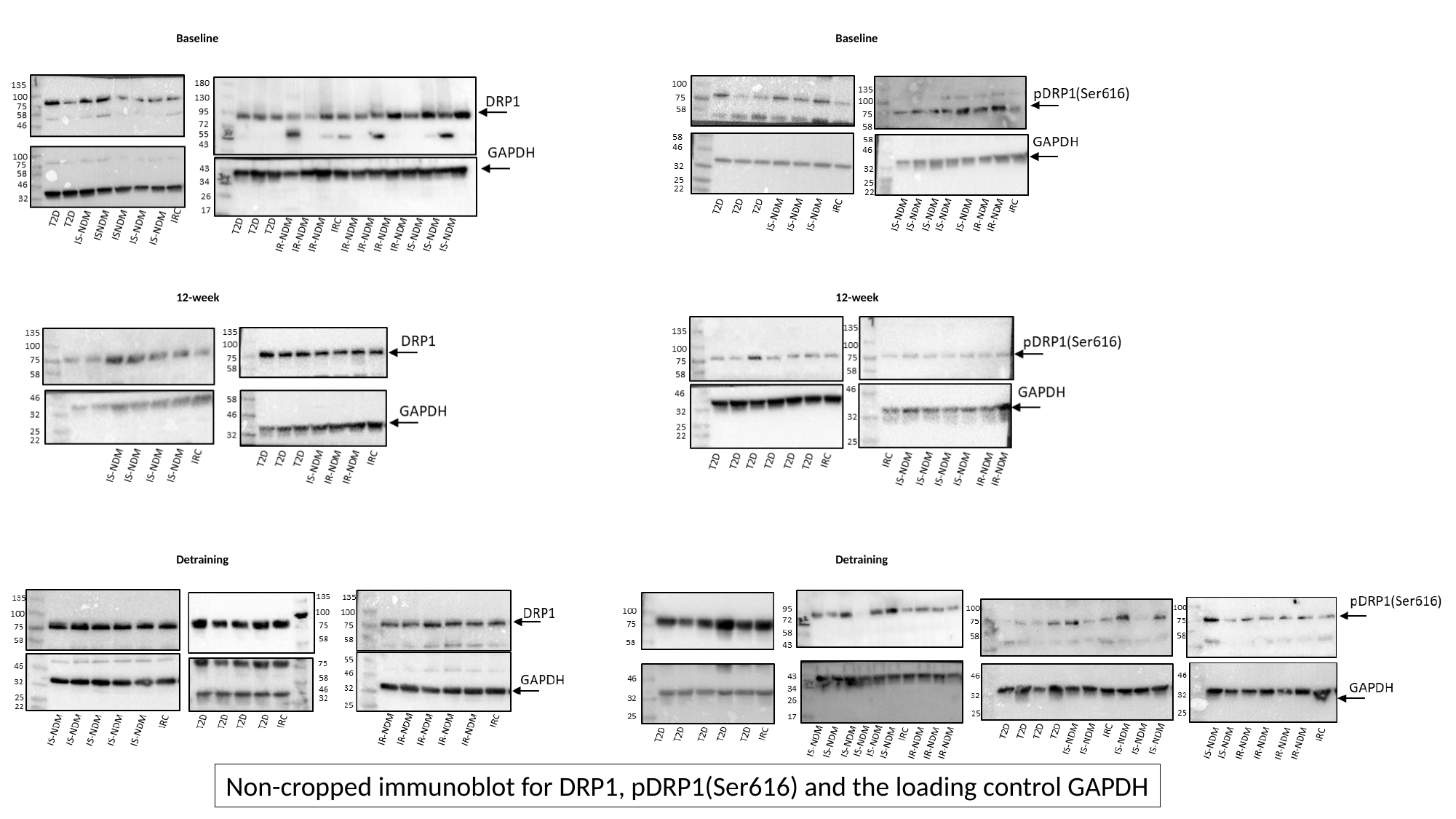

Baseline
Baseline
12-week
12-week
Detraining
Detraining
Non-cropped immunoblot for DRP1, pDRP1(Ser616) and the loading control GAPDH

## Slide 2
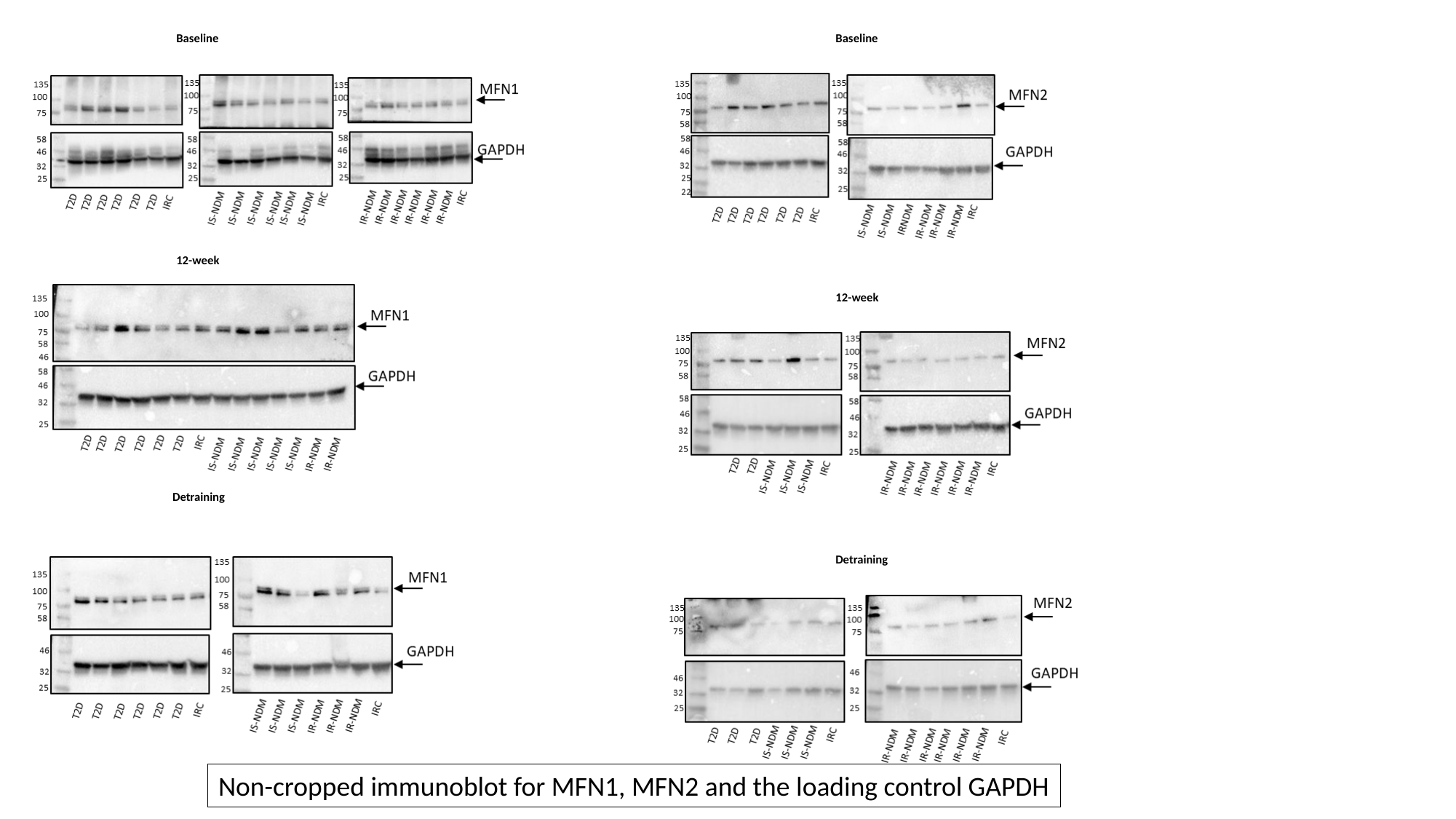

Baseline
Baseline
12-week
12-week
Detraining
Detraining
Non-cropped immunoblot for MFN1, MFN2 and the loading control GAPDH

## Slide 3
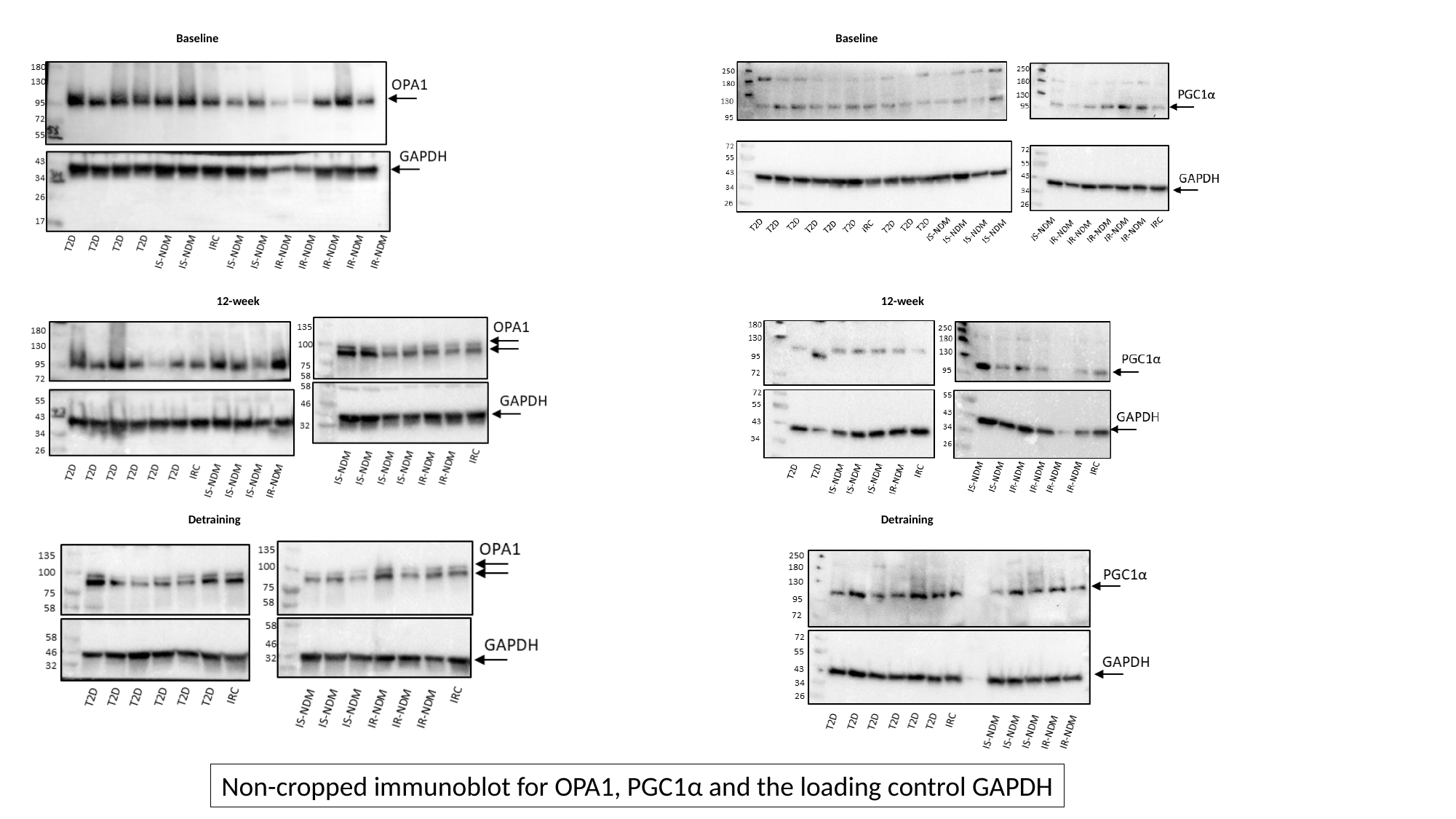

Baseline
Baseline
12-week
12-week
Detraining
Detraining
Non-cropped immunoblot for OPA1, PGC1α and the loading control GAPDH

## Slide 4
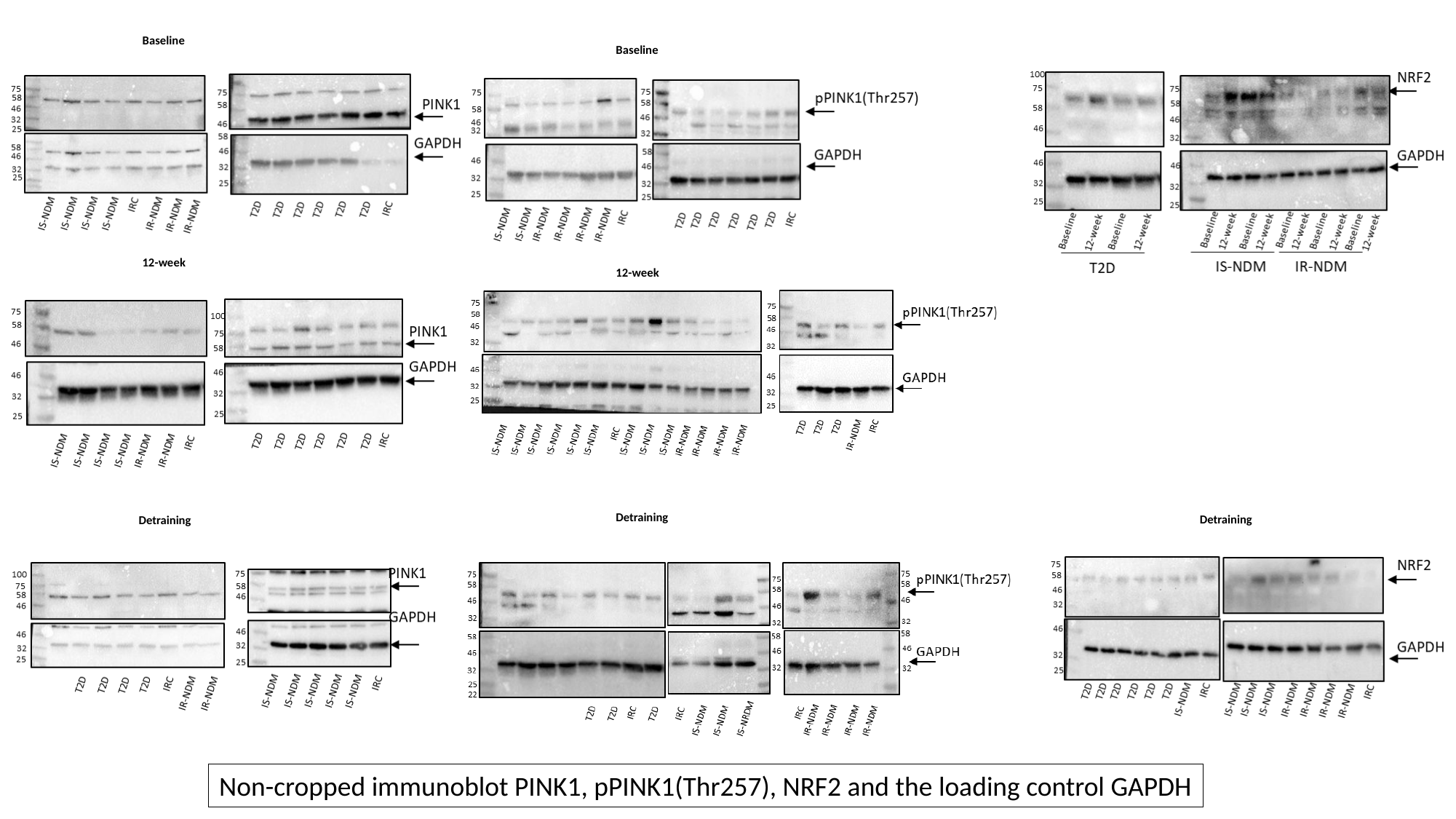

Baseline
Baseline
12-week
12-week
Detraining
Detraining
Detraining
Non-cropped immunoblot PINK1, pPINK1(Thr257), NRF2 and the loading control GAPDH

## Slide 5
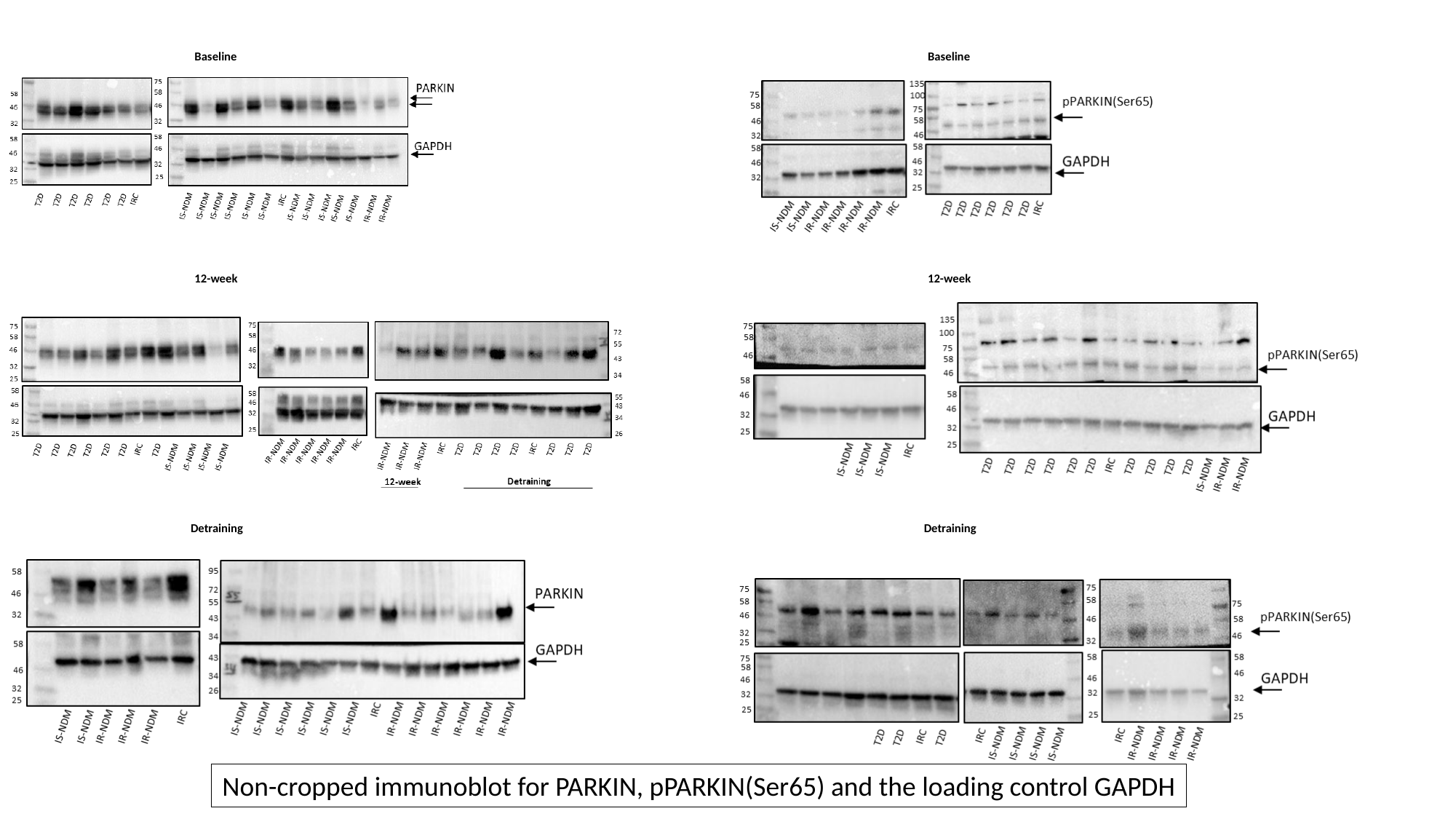

Baseline
Baseline
12-week
12-week
Detraining
Detraining
Non-cropped immunoblot for PARKIN, pPARKIN(Ser65) and the loading control GAPDH

## Slide 6
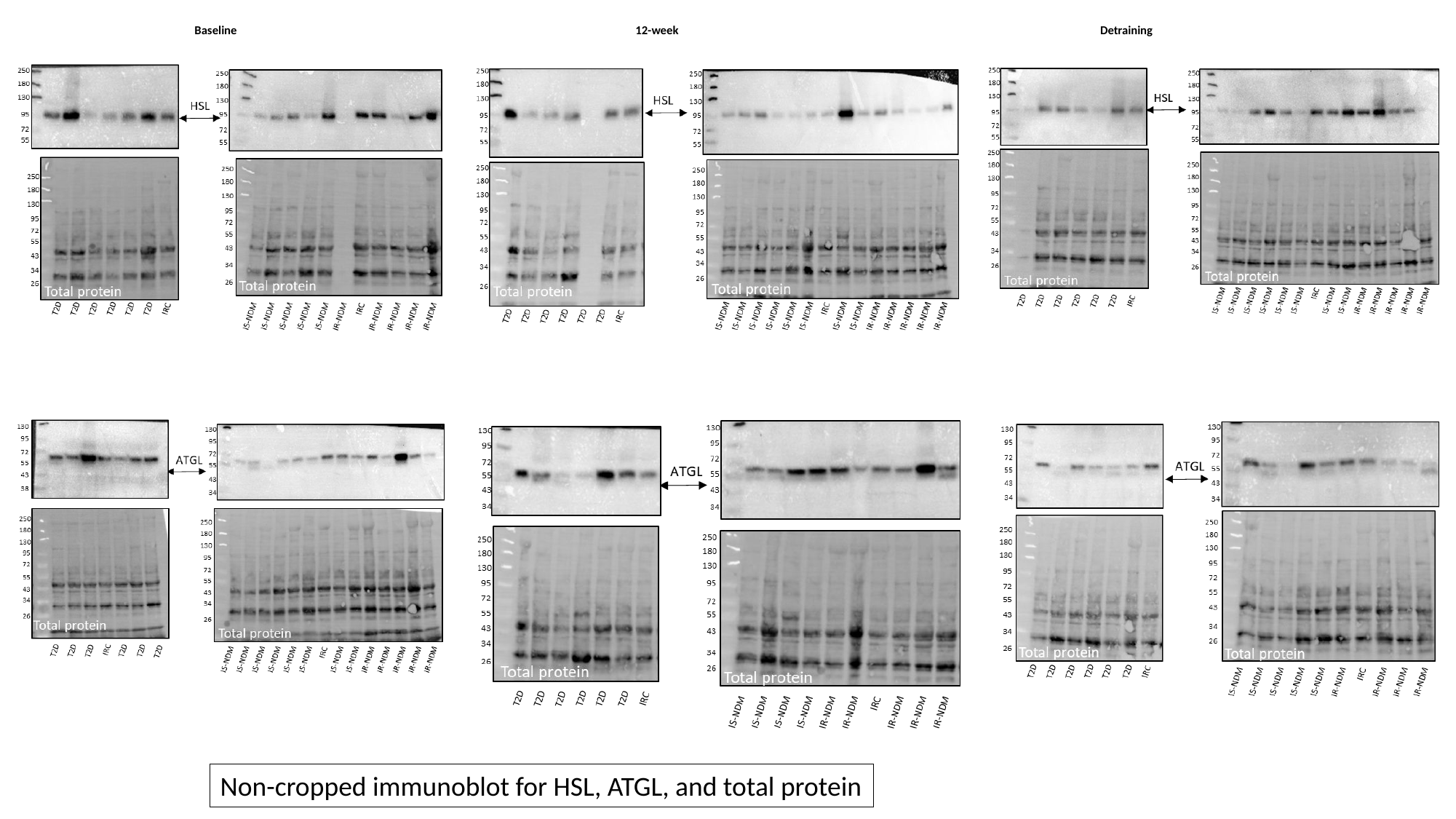

Baseline
12-week
Detraining
Non-cropped immunoblot for HSL, ATGL, and total protein

## Slide 7
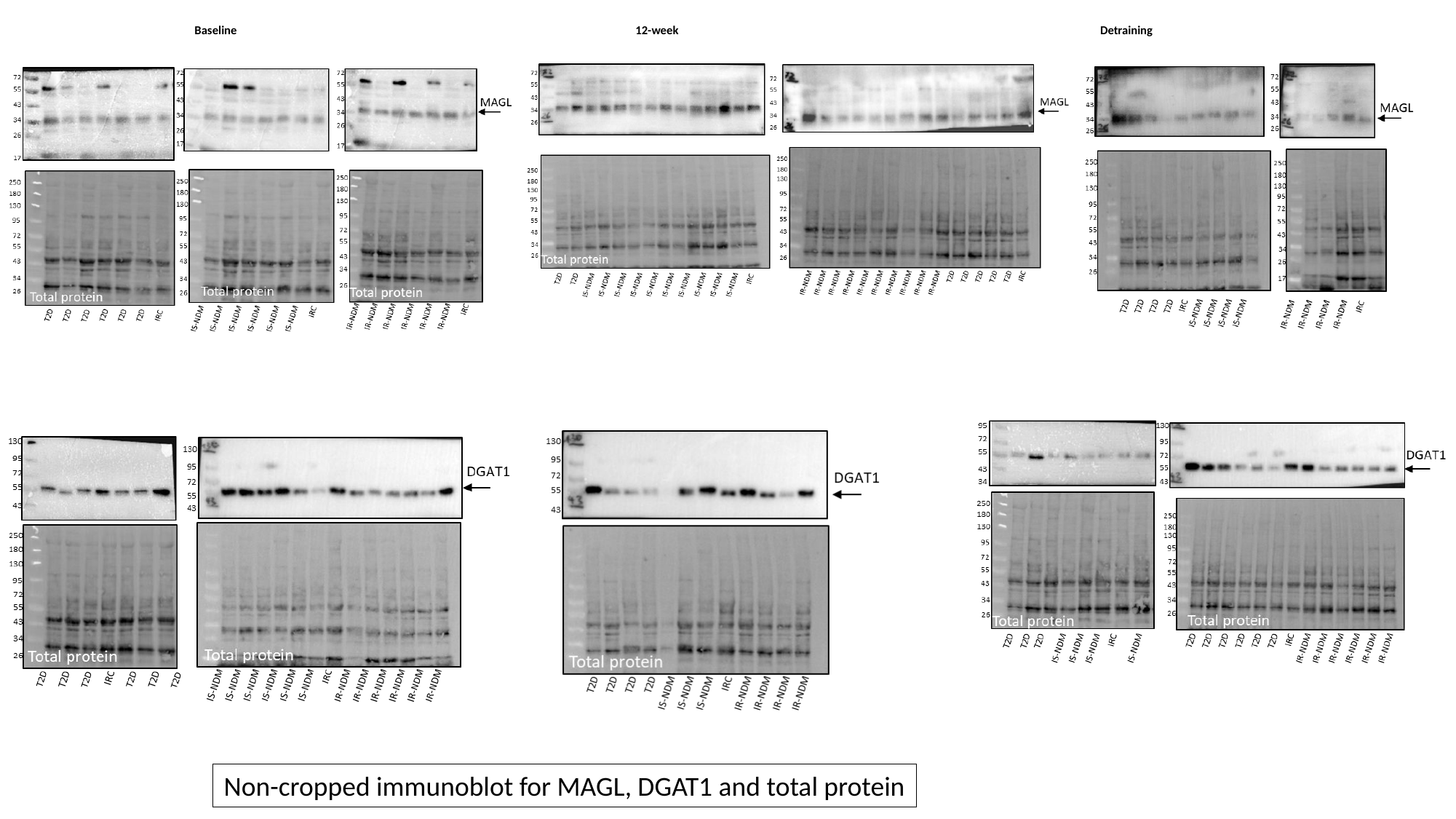

Baseline
12-week
Detraining
Non-cropped immunoblot for MAGL, DGAT1 and total protein
